# Development of Novel Chemically-Modified Nucleic Acid Molecules for Efficient Inhibition of Human *MAPT* Gene Expression

**DOI:** 10.3390/genes11060667

**Published:** 2020-06-19

**Authors:** Madhuri Chakravarthy, Suxiang Chen, Tao Wang, Rakesh N. Veedu

**Affiliations:** 1 Centre for Molecular Medicine and Innovative Therapeutics, Murdoch University, Perth 6150, Australia; m.chakravarthy@murdoch.edu.au (M.C.); S.Chen@murdoch.edu.au (S.C.); Tao.Wang@murdoch.edu.au (T.W.); 2 Perron Institute for Neurological and Translational Science, Perth 6150, Australia

**Keywords:** Alzheimer’s disease, *MAPT*, tau, DNAzymes, antisense oligonucleotides

## Abstract

The hyperphosphorylation of the microtubule-associated protein tau (MAPT) has been implicated in various neurological diseases, including Alzheimer’s disease. It has been hypothesized that the reduction of MAPT would result in depolymerizing neurofibrillary tangles and could be a potential strategy for the treatment of Alzheimer’s disease and other tauopathies. In this study, we report the development of novel DNAzymes and splice-modulating antisense oligonucleotides (AOs) for the efficient inhibition of MAPT. We designed and synthesized a range of DNAzymes and 2ʹ-O-methyl (2’-OMe)-modified AOs on a phosphorothioate (PS) backbone targeting various exons across the *MAPT* gene transcript. Our results demonstrated that RNV563, an arm-loop-arm-type DNAzyme targeting exon 13, and an AO candidate AO4, targeting exon 4, efficiently downregulated *MAPT* RNA expression by 58% and 96%, respectively. In addition, AO4 also reduced the MAPT protein level by 74%. In line with our results, we believe that AO4 could be used as a potential therapeutic molecule for Alzheimer’s disease and other tauopathies.

## 1. Introduction

Synthetic nucleic acid-based technologies offer great promise for the development of precision therapeutics for the treatment of various diseases. So far, nine oligonucleotide drug molecules have been approved for clinical use, including Vitravene for viral retinitis, Macugen for age-related macular degeneration, Kynamro for familial hypercholesterolemia, Exondys51 and Vyondys53 for Duchenne muscular dystrophy, Spinraza for spinal muscular atrophy, Onpattro and Tegsedi for hereditary transthyretin amyloidosis, and Waylivra for familial chylomicronemia syndrome [1,2]. Inspired by the rapid progress in the translation of nucleic acid drugs in recent years, we envisioned the development of novel nucleic acid molecules for targeting the microtubule-associated protein tau (*MAPT*), which is a protein that belongs to the microtubule-associated protein family [3]. The aggregation of hyperphosphorylated tau that leads to the formation of neurofibrillary tangles has been implicated in a variety of tauopathies, including Alzheimer’s disease (AD), frontotemporal dementia, Pick disease, argyrophilic grain disease, progressive supranuclear palsy, and corticobasal degeneration [3]. There are six isoforms of tau found in the adult brain produced through alternative splicing of the *MAPT* pre-mRNA [4,5] (Figure S9, Supplementary Material). Three of the isoforms have a three-repeat segment in the microtubule binding domain, while the other three have a four-repeat segment in the microtubule binding domain, and both groups are found in equal amounts in the adult brain [4,5].

**Figure 1:**
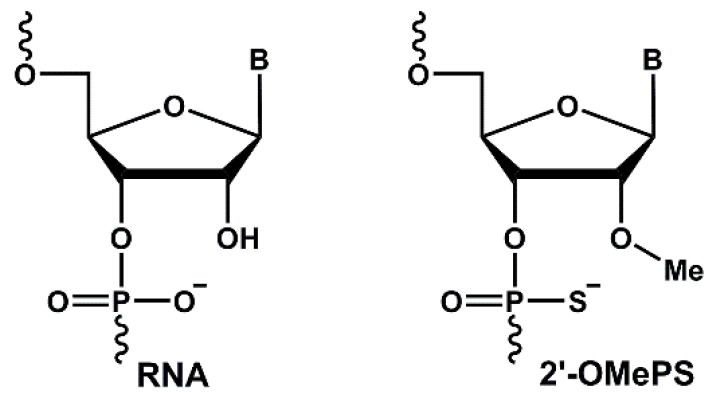
Structural representation of a 2’-O-methyl phosphorothioate (2’-OMePS) RNA monomer.

Tau levels are found to be elevated in the cerebrospinal fluid of AD patients. Although neurofibrillary tangles are a hallmark of AD, most drug development initiatives have targeted the amyloid beta pathway. However, due to the failure of drug molecules targeting the amyloid beta pathway, the focus is now shifting towards targeting hyperphosphorylated tau. One approach is the development of antisense oligonucleotides (AOs) against *MAPT* to reduce the production of hyperphosphorylated tau. A gapmer AO named *MAPT*_RX_ developed by IONIS Pharmaceuticals is currently being tested in Phase II clinical trials [6]. Another study evaluated phosphorodiamidate morpholino oligomers (PMOs) for downregulating tau expression in human neuroblastoma cells [7]. Although it is believed that tau plays a very important role in stabilizing microtubules (loss of this function has been attributed to neurodegeneration), mouse models have shown that tau knockout is well-tolerated and these mice only develop minor motor phenotypes at 12–16 months after tau knockout [8]. Furthermore, reducing tau levels in adult mice does not result in behavioral or neuroanatomical abnormalities, indicating that there may be other microtubule-associated proteins that can compensate for the loss of tau in microtubule stabilization [8,9]. Furthermore, it has been hypothesized that the reduction in tau monomers may result in depolymerization of the paired helical filaments to maintain equilibrium [9]. In this study, we developed novel DNAzymes and 2’-O-methyl (2’-OMe) phosphorothioate (PS) RNA-modified (Figure 1) steric blocking AOs targeting various *MAPT* exons for the efficient downregulation of tau protein levels.

DNAzymes are a unique class of oligonucleotides that have catalytic activity and can be used for various applications, including gene silencing, and in diagnostic tools as biosensor molecules [10,11,12,13]. DNAzymes bind to their complementary mRNA targets through Watson–Crick base pairing. They possess catalytic activity, through which they degrade their target by cleavage of the phosphodiester bond at the purine–pyrimidine junction or at the purine–purine junction [14,15,16,17]. DNAzymes require divalent metal ions as cofactors for their catalytic activity similar to naturally occurring enzymes [14]. Unlike RNase-H-dependent AOs, DNAzymes do not require RNase-H recruitment for target mRNA cleavage.

Steric-blocking AOs bind to the target mRNA and can block the access of cellular machinery to pre-mRNA and mRNA, and can therefore modulate splicing, repair defective RNA, restore protein production, or downregulate gene expression without degrading the RNA [18,19,20]. Steric-blocking AOs that modulate splicing have been approved for clinical use for the treatment of Duchene muscular dystrophy and spinal muscular atrophy [21,22]. Splice-modulating AOs bind to their complementary mRNA targets through Watson–Crick base pairing and modulate splicing through blocking the interaction of the pre-mRNA with splicing factors such as RNA-binding proteins, small nuclear RNAs, and other components of the spliceosome [23]. 

## 2. Materials and Methods 

### 2.1. DNAzymes and AOs

DNAzymes with stem-loop conformation were designed for the selected exons (Table S1, Supplementary Material), in line with our previous work on antimiRzymes [24], and the oligonucleotides were ordered from Integrated DNA Technologies (IDT, Singapore). AOs (uniformly 2’-OMePS modified) were designed and synthesized for the selected exons, in line with our previous work on Duchenne muscular dystrophy (DMD) [25,26,27,28,29,30,31]. The DNAzymes and 2’-OMePS AOs were designed to target the selected exons of *MAPT* variant 2, which is the longest isoform found in the adult brain. The 2’-OMePS AOs were synthesized in-house on the AKTA Oligopilot 10 synthesizer (GE Healthcare Life Sciences, Chicago, USA) using standard phosphoramidite chemistry at a 1 µmole scale (AO names and their sequences are listed in Table S3, Supplementary Material). The synthesized AOs were deprotected by treatment with 1 mL ammonium hydroxide (Sigma, Castle Hill, Australia; Cat# 221228-500mL) overnight at 55 °C and were purified by desalting using the illustra NAP-10 columns (GE Healthcare; Cat# 45-000-153), according to the manufacturer’s protocol and verified by HPLC.

### 2.2. Cell Culture and Transfection of AOs and DNAzymes

Cell culture media and supplements were purchased from Life technologies, Australia, unless specified. SH-SY5Y cells were obtained from Cell Bank Australia (kindly provided by Prof. Sue Fletcher and Prof. Steve Wilton). SH-SY5Y cells were propagated in Dulbecco’s modified Eagle’s medium: F12 medium and 10% fetal bovine serum (Serana, Wembley, Australia; Cat# FBS-AU-015) in a humidified atmosphere in a 37 °C incubator with 5% CO_2_. Cells were maintained at 70–90% confluency and seeded onto a plate or flask for transfections. Transfections were performed in 24 well-plate formats with approximately 70,000 cells/well for RNA transcript analysis. Transfections were also performed in T25 cm^2^ format with approximately 875,000 cells/well for protein analysis. The cells were seeded one day before being transfected with the DNAzymes or 2’-OMePS AOs complexed with Lipofectamine 3000 transfection reagent (ThermoFisher Scientific, Malaga, Australia; Cat# L3000015), as per the manufacturer’s protocol. Transfection was carried out for 24 h before harvesting RNA for transcript analysis. For protein analysis, transfection was carried out 24 h, 48 h (re-transfected at 24 h), and six days (re-transfected at 24 and 48 h) before collecting the cell pellet. 

### 2.3. RT-PCR Analysis of AOs and DNAzymes Treatment

Total RNA was extracted using the Bioline Isolate II RNA MiniPrep Kit (Bioline, Eveleigh, Australia; Cat# BIO-52073), following the manufacturer’s protocol. A total of 50 ng of total RNA was analysed using primer sets (ordered from IDT) listed in Table S4 of Supplementary Material, in the Superscript III One-Step RT-PCR System (ThermoFisher Scientific; Cat# 12574026), and the reaction conditions are given in Table S5 of Supplementary Material. The primers used to perform the RT-PCR were designed to amplify the longest *MAPT* isoform—variant 2. GAPDH was used as a loading control and the primer set (ordered from IDT), and RT-PCR conditions for GAPDH are given in Tables S4 and S5 of Supplementary Material. The products were separated on a 2% agarose gel in Tris-acetate-EDTA buffer, stained with Red Safe (iNtRON Biotechnology, Seongnam, South Korea; Cat# 21141), and destained with water, before being visualized with the Fusion Fx gel documentation system (Vilber Lourmat, Collégien, France).

### 2.4. Densitometry Analysis

Densitometry (measuring the band intensity) of the bands from the gels was performed using Image J Software [32]. The intensity of the *MAPT* bands (longest *MAPT* isoform, variant 2) in different DNAzyme- or AO-treated samples was measured and normalized to the intensity of the corresponding GAPDH bands, before being compared to the band intensity of the scrambled samples or untreated samples. The percentage of the longest *MAPT* isoform, calculated as variant 2 transcript knockdown in SH-SY5Y cells, was expressed as the activity of DNAzyme or AO. All percentages given in this study refer to the longest *MAPT* isoform—variant 2.

### 2.5. In Vitro Cleavage Assay Using DNAzymes

The cleavage efficiency of the second-generation DNAzymes in vitro was tested using the in vitro cleavage assay. A synthetic fluorescein dye (FAM)-labeled RNA target specific to the exon 11 region of the *MAPT* transcript was used. The experiments were performed by incubating DNAzymes RNV563 and their second-generation variants with an FAM-labeled RNA template in the presence of divalent metal ions, and the products were separated and analysed on a denaturing polyacrylamide gel. A total of 1.76 µM DNAzymes was incubated with an equal molar concentration of FAM-conjugated *MAPT* RNA (5′-FAM-UGUGGCUCAUUAGGCAACAUCCAUCAUAAACCAG-3′) in 5 µL of buffer containing Mg^2+^ divalent cations (10 mM MgCl_2_) at 37 °C. The reaction was stopped by adding 10 µL of formamide to 10 µL of the reaction mixture at 0 min, 30 min, 60 min and 2 h. Scrambled DNAzyme RNV563-SCR (AACATCCTCGTTGTAGCTAGCCTCATAAAC), RNV563-Mut1 (GTTTATGAAACTAGCTACAACGAGGATGTT), and RNV563-Mut2 (GTTTATGAGGCTAAATACAACGAGGATGTT) were used as negative controls, and the untreated samples did not have any DNAzyme. The underlined bases are the mutated bases and the two double mutants RNV563-Mut1 and RNV563-Mut2 were designed based on previous papers [33,34]. The reaction mixtures were separated on a 15% polyacrylamide gel/7M urea for 50 mins at 13 W. The gel was visualized using the Fusion Fx gel documentation system (Vilber Lourmat, Collégien, France). The cleavage of the 34mer full-length FAM-conjugated RNA target is expected to yield 22 nucleotide-long products. 

### 2.6. Western Blot

Western blot analysis was performed on the best performing AO to evaluate the effect of the AO on the inhibition of the *MAPT* protein in comparison to the scrambled AO-treated and untreated samples. Frozen transfected cell pellets were thawed and homogenized in SDS lysis buffer (0.5 M Tris-HCL pH 6.8, 3% SDS (*w*/*v*), and 10% glycerol (*v*/*v*)) containing protease inhibitor (Sigma, Castle Hill, Australia). The proteins were separated on a 10% polyacrylamide gel containing 400 mM Tris-HCL (pH 8.8) and 0.1% SDS in a Tris-glycine-SDS running buffer, before being transferred to a 0.2 µm nitrocellulose membrane (Biorad, Gladesville, Australia; Cat# 162-0112) in a Tris-glycine-methanol transfer buffer. The membranes were blocked in 5% skim milk Tris-buffered saline with 0.1% Tween. The membrane was washed in Tris-buffered saline with 0.1% Tween, and incubated with primary antibodies overnight at 4 °C, 1:2000 anti-total tau (Abcam, Cambridge, UK; Cat# ab76128), and 1:1000 anti-GAPDH (ThermoFisher Scientific, Malaga, Australia; Cat# PA1-988). After washing the membrane in Tris-buffered saline with 0.1% Tween, the membrane was incubated with the secondary antibody (1:5000 anti-rabbit HRP, Thermofisher Scientific, Malaga, Australia; Cat# 31460) for 1 h at room temperature before washing. The antibodies were detected using a Clarity Western ECL detection kit (Biorad, Gladesville, Australia; Cat# 1705060) according to the manufacturer’s protocol and visualized using a chemiluminescence-based protocol on the Fusion Fx gel documentation system (Vilber Lourmat, Collégien, France).

## 3. Results

### 3.1. Design and Screening of DNAzymes Targeting MAPT mRNA

**Figure 2:**
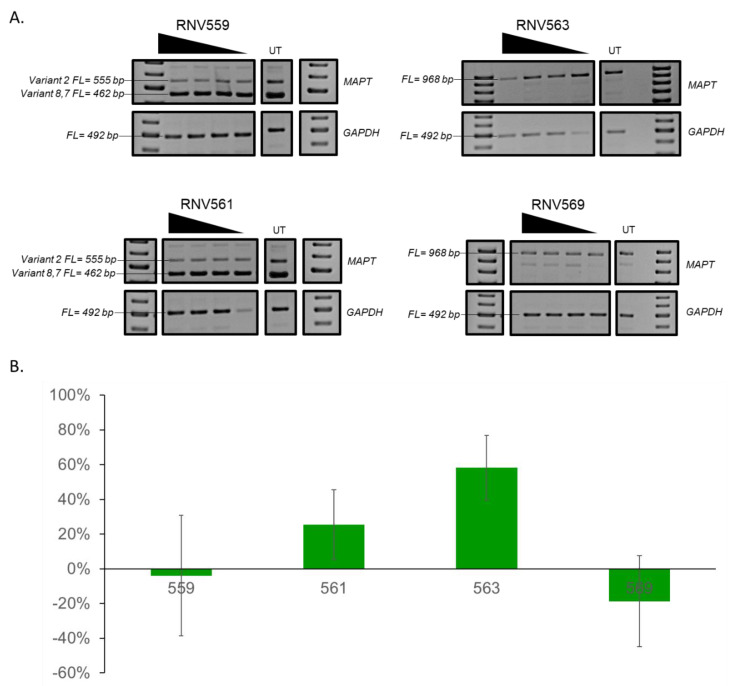
RT-PCR analysis of DNAzymes RNV559, RNV561, RNV563, and RNV569 treated SH-SY5Y cells. (**A**) Representative RT-PCR products of the microtubule-associated protein tau (*MAPT*) and *GAPDH* transcripts from SH-SY5Y cells after treatment with DNAzyme RNV559, RNV561, RNV563, and RNV569 at 400, 200, 100, and 50 nM concentrations. (**B**) The average activity of first-generation DNAzymes (at a 400 nM concentration) in SH-SY5Y cells (knockdown of the *MAPT* transcript). The average activity was obtained through three replicates. The error bars represent the standard error of the mean. The RNA from RNV559- and RNV561-treated samples was analysed using primer set 3 and the RNA from RNV563- and RNV569-treated samples was analysed using primer set 4. FL, full-length; UT, untreated. *GAPDH* was used as a loading control. The gel images were cropped to highlight the *MAPT*-specific products and the corresponding house-keeping gene control *GAPDH*. The original images are shown in Figure S2 (Supplementary Material).

Arm-loop-arm-type DNAzymes with a 10–23 catalytic motif were designed to target various exons of the *MAPT* transcript (Table S1, Supplementary Material). The sequences of the catalytic regions were pre-fixed according to previous reports [35,36] and the arm regions were designed to be specific and complementary to the *MAPT* mRNA sequences. Initially, the catalytic activities of all the designed DNAzymes against *MAPT* mRNA were screened in SH-SY5Y neuroblastoma cells by transfecting at two different concentrations (400 and 50 nM) for 24 h. Total RNA was extracted from the cell lysate, and the integrity of the *MAPT* transcript was assessed by performing RT-PCR. Dose-dependent reduction of the full-length *MAPT* transcript was observed at various levels for RNV559, RNV561, RNV563, RNV569, RNV570, and RNV571 DNAzyme candidate-treated samples (Figure S1, Supplementary Material). The best *MAPT* transcript knockdown was observed for RNV559, RNV561, RNV563, and RNV569 DNAzymes at 400 nM. The catalytic activities of RNV559, RNV561, RNV563, and RNV569 were further validated against *MAPT* mRNA in SH-SY5Y cells at four different concentrations, including 50, 100, 200, and 400 nM (Figure 2). DNAzyme candidate RNV563 targeting the exon 11 of the *MAPT* mRNA showed the highest efficacy, with 58% knockdown of *MAPT* mRNA, followed by RNV561 (26%) (Figure 2A; densitometry analysis in Figure S2B of Supplementary Material). 

### 3.2. Design and Screening of Second-Generation DNAzymes Targeting MAPT mRNA

Based on the initial screening outputs, the best performing DNAzyme RNV563 was selected for further modifications in order to improve the efficacy by systematically increasing and decreasing the binding arm lengths. Many studies have shown that modifying the length of the hybridization arms on either side of the catalytic motif can increase the binding affinity and efficacy [37,38,39]. In our study, the first generation of DNAzymes initially had eight nucleotides on one arm and seven on the other. Several studies have shown that the optimal arm lengths vary from 7 to 10 nucleotides long. Therefore, the length of RNV563 was increased by one to three nucleotides at the end of both arms and named RNV610, RNV611, and RNV612 (Table 1), and the efficacy of the modified DNAzymes was then verified. Additionally, we decreased the arm length of our first-generation DNAzyme RNV563 and verified the efficacy of the shorter DNAzymes by removing one and two nucleotides from both ends and naming them RNV608 and RNV609 (Table 1). The catalytic activities of these second-generation DNAzyme candidates were analysed in SH-SY5Y as described above. The transfections were repeated three times. Decreasing the length of the RNV563 completely abolished the DNAzyme activity of RNV608 (0%) and RNV609 (0%), even at 600 nM, compared to the parent DNAzyme RNV563 (56%). Furthermore, increasing the length of the RNV563 also reduced the DNAzyme activity of RNV610 (11%), RNV611 (1%), and RNV612 (6%) (Figure S3 and Table S2, Supplementary Material). These results showed that increasing and decreasing the arm length abolished the efficacy of DNAzyme RNV563 in SH-SY5Y cells.

### 3.3. In Vitro Cleavage of the MAPT RNA Template

**Figure 3:**
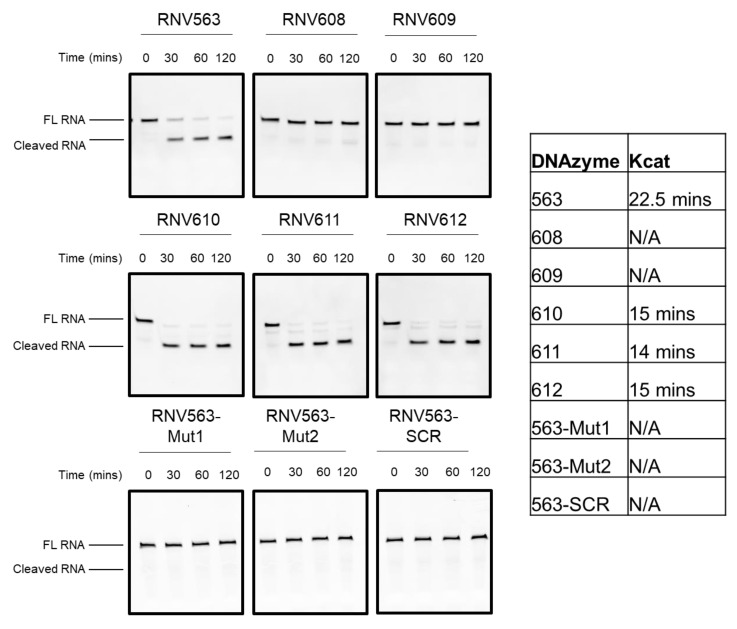
In vitro cleavage of the synthetic fluorescein dye (FAM)-conjugated *MAPT* RNA template by RNV563 and its derivatives. The table provides the catalytic activity. FL RNA, full-length FAM-conjugated RNA; cleaved RNA, cleaved FAM-conjugated *MAPT* RNA (22 nucleotides long). The FAM-conjugated template RNA is a small region of the *MAPT* transcript complementary to the hybridization arms of the DNAzymes. The gel images were cropped for a better overview. The original images are shown in Figure S4 (Supplementary Material).

To further verify and understand the catalytic activity of DNAzymes targeting the *MAPT* transcript, we tested the cleavage efficacy in vitro using a synthetic fluorescein dye (FAM)-labeled RNA target specific to the exon 11 region of the *MAPT* transcript. The experiments were performed by incubating DNAzymes RNV563 and their second-generation variants with an FAM-labeled RNA template in the presence of divalent metal ions, and the products were separated and analysed on a denaturing polyacrylamide gel. Briefly, 1.76 µM DNAzymes was incubated with 1.76 µM FAM-conjugated *MAPT* RNA in the presence of Mg^2+^ for 30, 60, and 120 min at 37 °C. The reactions were stopped by adding 10 µL of formamide solution. The products were then separated on a 15% denaturing polyacrylamide gel and visualized using the Fusion Fx gel documentation system (Vilber Lourmat). The cleavage of the 34mer full-length FAM-conjugated RNA target is expected to yield 22 nucleotide-long products. A scrambled (SCR) sequence and RNV563 mutants with different mutations (RNV563Mut1 and RNV563Mut2) within the catalytic region of RNV563 were used as negative controls in parallel and an untreated (UT) sample with no DNAzyme was also included. DNAzymes RNV563, RNV610, RNV611, and RNV612 showed efficient cleavage of the *MAPT* RNA template in vitro, whereas the RNV608 and RNV609 with decreased arm lengths failed to yield any RNA cleavage (Figure 3). Notably, the in vitro cleavage rates of the DNAzymes with increased arm lengths, RNV610, RNV611, and RNV612, were faster than that of their parent DNAzyme, RNV563. As expected, the scrambled control and the mutant RNV563 did not show any cleavage (Figure 3).

### 3.4. Evaluation of Splice Modulating AOs to Induce Exon-Skipping in the MAPT Transcript 

**Figure 4:**
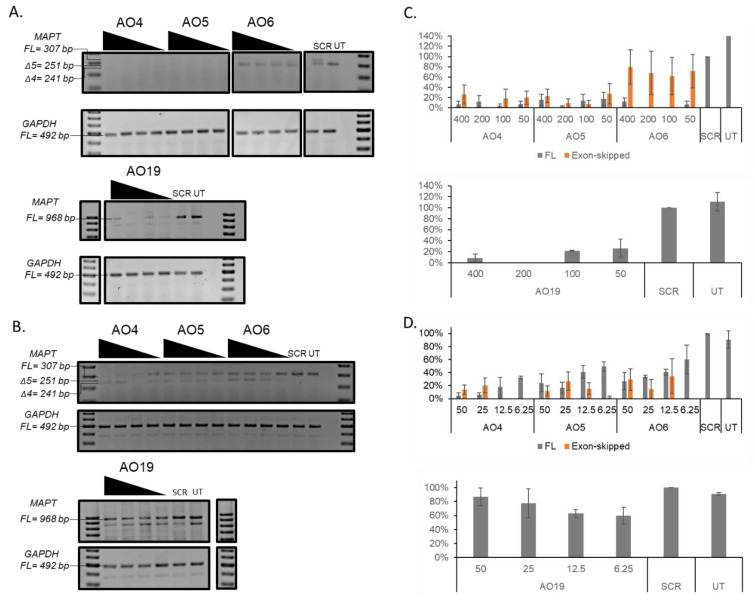
(**A**) Representative RT-PCR products of the *MAPT* and *GAPDH* transcripts from SH-SY5Y cells after treatment with AO4, AO5, AO6, and AO19 at 400, 200, 100, and 50 nM concentrations. Treatment with a scrambled sequence (SCR) antisense oligonucleotide (AO) was at 400 nM. (**B**) Representative RT-PCR products of the *MAPT* and *GAPDH* transcripts from SH-SY5Y cells after treatment with AO4, AO5, AO6, and AO19 at 50, 25, 12.5, and 6.25 nM concentrations. Treatment with SCR AO was at 50 nM. (**C**) Densitometry analysis of RT-PCR products (three replicates) normalized to the SCR control using AO4, AO6, AO7m and AO19 showed downregulation and exon-skipping of the *MAPT* transcript in SH-SY5Y cells in vitro. Concentrations of AOs used include 400, 200, 100, and 50 nM, with an SCR concentration of 400 nM. The error bars represent the standard error of the mean. (**D**) Densitometry analysis of RT-PCR products (three replicates) normalized to the SCR control using AO4, AO6, AO7, and AO19 showed downregulation and exon-skipping of the *MAPT* transcript in SH-SY5Y cells in vitro. Concentrations of AOs used include 50, 25, 12.5, and 6.25 nM, with an SCR concentration of 50 nM. The error bars represent the standard error of the mean. AO4 targets exon 4, AO5 and AO6 target exon 5, and AO19 targets exon 12 of the *MAPT* transcript. RNA from AO4-, AO5-, and AO6-treated samples was analysed using primer set 2. RNA from AO19-treated samples was analysed using primer set 4. FL, full-length; UT, untreated; SCR, scrambled sequence. *GAPDH* was used as a loading control. The gel images were cropped to highlight the *MAPT*-specific products and the corresponding house-keeping gene control *GAPDH*. The original images are shown in Figure S6 (Supplementary Material).

Firstly, we evaluated the exon-skipping efficiency of all AOs targeting different exons (Table S3, Supplementary Material) in vitro at two different concentrations (400 and 50 nM). The results demonstrated that AO4, AO5, AO6, and AO19 targeting exons 4, 5, and 19 resulted in exon-skipping and downregulation of the *MAPT* transcript in vitro (Figure S5, Supplementary Material). AO4, AO5, AO6, and AO19 were further validated at 400, 200, 100, and 50 nM (Figure 4A; see Figure 4C for densitometry analysis). The results showed that AO4 (92%), AO5 (83%), AO6 (93%), and AO19 (74%) were all efficient at downregulating *MAPT* transcript levels (Figure 4A,C), even at the lowest concentration (50 nM) tested, compared to the scrambled control. AO4 (20%) and AO5 (27%) induced *MAPT* exon 4 skipping. AO6 treatment of SH-SY5Y cells resulted in *MAPT* exon 5 skipping (71%) at a 50 nM concentration (Figure 4A,C). The AO4, AO5, AO6, and AO19 were further tested at lower concentrations, including 50, 25, 12.5, and 6.25 nM, with *GAPDH* as a loading control as they showed downregulation of the *MAPT* transcript at the higher concentrations (Figure 4B; see Figure 4D for densitometry analysis). Notably, AO4 (20%) and AO5 (27%) induced *MAPT* exon 4 skipping and were most efficient at 25 nM. In addition, AO4 (96%) and AO5 (76%) also resulted in dose-dependent downregulation of the *MAPT* transcript and were most efficient at 50 nM. Although AO19 downregulated the *MAPT* transcript, it failed to demonstrate dose-dependent inhibition. AO4 was further validated in inhibiting the expression of the *MAPT* protein as it was found to be the most efficient candidate at inhibiting the *MAPT* transcript.

### 3.5. Evaluation of MAPT Protein Downregulation Using AO4

**Figure 5:**
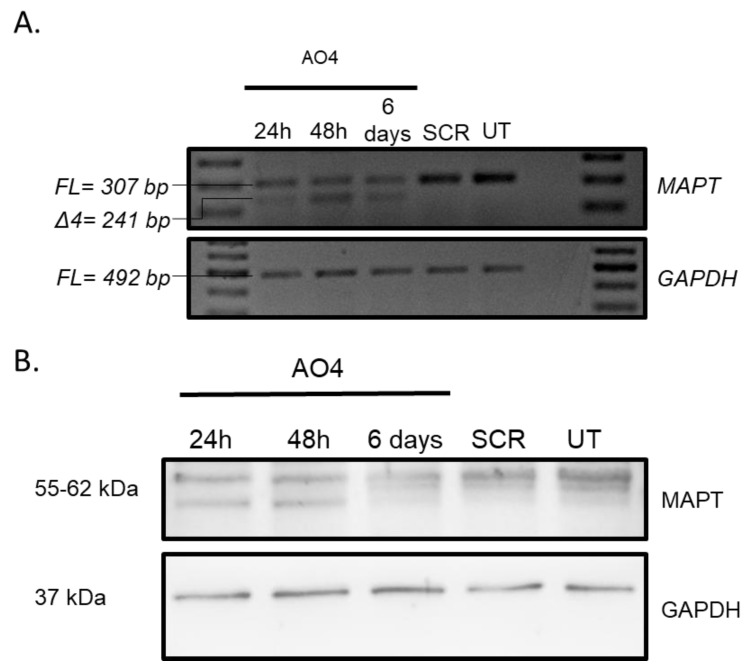
(**A**) Representative RT-PCR products of the *MAPT* transcripts from SH-SY5Y cells after treatment with an AO4 50 nM concentration and incubation of AO for 24 h, 48 h, and six days. RNA from AO4-treated samples was analysed using primer set 2. (**B**) Representative protein products of the *MAPT* and *GAPDH* transcripts from SH-SY5Y cells after treatment with AO4 at 50 nM concentrations and incubation of AO for 24 h, 48 h, and six days. RT-PCR products of the *MAPT* transcripts and protein products of the *MAPT* and *GAPDH* transcripts from SH-SY5Y cells after treatment with SCR at 50 nM concentrations and incubation of SCR for six days. FL, full-length; UT, untreated; SCR, scrambled sequence. GAPDH was used as a loading control. The gel images were cropped to highlight the *MAPT*-specific products and the corresponding house-keeping gene control GAPDH. The original images are shown in Figure S7 (Supplementary Material).

**Figure 6:**
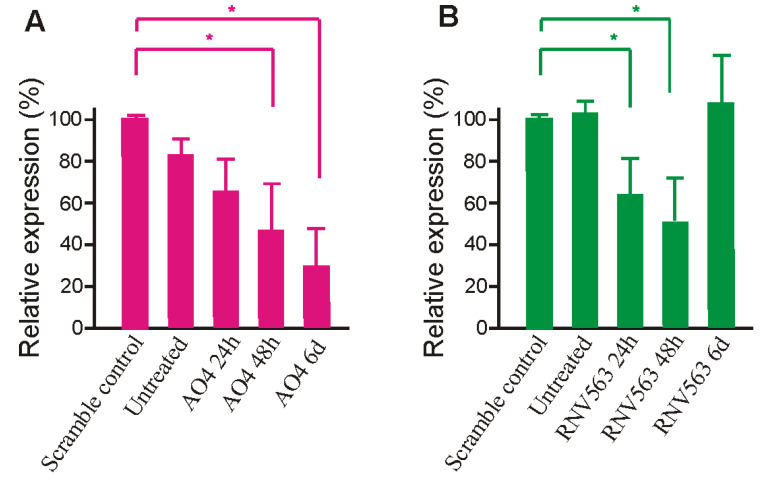
(**A**) Densitometry analysis of the tau expression obtained from western blot (three replicates) analysis after the treatment of SH-SY5Y cells with AO4 at 50 nM concentrations and incubation of AO4 for 24 h, 48 h, and six days. SH-SY5Y cells were treated with the scrambled control at a 50 nM concentration and incubation of six days. AO4 targets exon 4 of the *MAPT* transcript. The error bars represent the standard error of the mean. (**B**) Densitometry analysis of the tau expression obtained from western blot (three replicates) analysis after the treatment of SH-SY5Y cells with RNV563 at 400 nM concentrations and incubation of DNAzyme for 24 h, 48 h, and six days. SH-SY5Y cells were treated with the scrambled control at a 400 nM concentration and incubation of six days. RNV563 targets exon 9 of the *MAPT* transcript. The error bars represent the standard error of the mean.

To further evaluate the efficacy of AO4 and RNV563 in inhibiting *MAPT* expression, the total *MAPT* protein level was evaluated in SH-SY5Y cells after 24 h, 48 h, and six days of transfection with AO4 and RNV563. Briefly, AO4- and RNV563-treated SH-SY5Y cells were incubated for 24 h, 48 h, and six days before collection. The cell pellet was collected and lysed, and the proteins were separated on a 10% separating gel and transferred onto a nitrocellulose membrane. The membrane was incubated with 1:2000 dilution of the *MAPT* (total tau) antibody overnight at 4 °C. The results clearly demonstrated that there was downregulation of the *MAPT* gene transcript and *MAPT* protein in cells 24 h, 48 h, and six days after AO4 treatment; however, the highest *MAPT* protein downregulation (74%) was seen six days after transfection (Figure 5 and Figure 6A for densitometry analysis). The results also demonstrated that there was downregulation of the *MAPT* protein in cells 24 and 48 h after RNV563; however the *MAPT* protein was close to *MAPT* protein levels in untreated and scrambled control-treated cells after six days of incubation. The highest *MAPT* protein downregulation (50%) was seen 48 hours after RNV563 transfection (Figure 6B for densitometry analysis, Figure S9B, Supplementary Material for western blot analysis). 

## 4. Discussion

Hyperphosphorylated tau-based neurofibrillary tangle formation is believed to be a pathological hallmark for various tauopathies, including Alzheimer’s disease (AD). Although some early work focused on developing nucleic acid-based *MAPT* inhibitors, we envisioned revisiting this target for the development of novel nucleic acid molecules for efficient tau inhibition. This study screened various DNAzymes and AOs targeting different *MAPT* exons of the longest *MAPT* isoform—variant 2. The primers used in this study for RT-PCR analysis only amplify certain *MAPT* variants (shown in Figure S9, Supplementary Material). Therefore, this study aimed to study the effects of the DNAzymes and AOs on the longest *MAPT* isoform, variant 2, which contains all of the exons and can thus be amplified by all of the primers used in this study. The effects on other variants can only present a partial picture, as not all primer sets amplify all six variants found in the adult brain. The *MAPT* transcripts detected by all of the primer sets used in this study are given in Table S6 (Supplementary Material). We screened various DNAzymes targeting different *MAPT* exons at two concentrations in SH-SY5Y cells (Figure S1, Supplementary Material) and the four identified candidates RNV559 (targeting exon 9), RNV561 (targeting exon 9), RNV563 (targeting exon 11), and RNV569 (targeting exon 13) efficiently downregulated the *MAPT* transcript. Further systematic evaluation at 400, 200, 100, and 50 nM concentrations identified RNV563 as the most efficient DNAzyme candidate. The RT-PCR full-length product of the RNV563-treated sample was sequenced and was demonstrated to align with the longest *MAPT* isoform, variant 2 (Figure S10, Supplementary Material), confirming that RNV563 downregulates the longest *MAPT* isoform. The variable efficiencies of downregulating the *MAPT* transcript observed in this study were similar to those observed in previous studies, including our work on DNAzymes targeting various regions of the *ITGA4* mRNA [34], and others by Vester et al. and Kurreck et al [40]. These studies have suggested that the DNAzyme’s catalytic efficiency for cleaving its target may vary due to its dependence on the RNA’s tertiary structure. The RNA tertiary structure affects the accessibility of the purine-pyrimidine cleavage sites to the DNAzyme and may differ between different regions of the mRNA, which may explain the observed differences in cleavage efficiencies. 

The optimal arm length, the symmetry or asymmetry of the arms, and the GC content may all affect the catalytic efficiency of the resulting DNAzyme, although how they affect the DNAzyme activity is unclear. The target binding arm of the DNAzyme is an important factor in determining the cleavage efficiency. Similar to our previously reported *ITGA4*-targeting DNAzyme RNV143 [34], we designed DNAzymes targeting *MAPT* with asymmetrical arms. The most efficient first-generation DNAzyme RNV563 was selected for further analysis by increasing and decreasing the arm lengths based on the cleavage efficiency. Decreasing the arm length of DNAzyme RNV563 resulted in a decreased cleavage efficiency in both SY-SY5Y cells and in the in vitro cleavage assay. The decreased cleavage efficiency observed could be due to the decreased binding affinity of the shorter arm lengths of the DNAzymes to their target RNA. In contrast, increasing the arm length of DNAyzme RNV563 resulted in a reduced cleavage efficiency in SH-SY5Y cells, but improved cleavage efficiency of the synthetic RNA in the in vitro cleavage assay. Increasing the arm lengths of the DNAzyme RNV563 may increase the binding affinity to the target RNA, leading to an increased cleavage efficiency of the synthetic RNA in vitro; however as shown by a previous study, cleavage of the shorter unstructured RNA is more efficient than longer structured RNA and therefore, the in vitro cleavage efficiency may not be a good representative of the in-cell environment and the synthetic RNA used in the in vitro assay may not be a good representative of the longer and more complex structure of the RNA naturally found in cells [37,41] Furthermore, an increased binding affinity may also lead to slower product release that would inhibit or decrease the rate of multiple turnovers. Therefore, increasing the arm length of the DNAzyme RNV 563 may have increased the binding affinity and decreased the rate of multiple turnovers, and thus resulted in the decreased cleavage efficiency observed in SH-SY5Y cells. This effect may not have been replicated in the in vitro cleavage assay as the amount of synthetic RNA used for the in vitro cleavage assay is limited. Therefore, an accurate picture on the effect of increasing the arm length of the DNAzyme on the rate of multiple turnovers may not be provided. 

We also identified an exon-skipping AO that efficiently downregulates the *MAPT* transcript in this study. AOs were designed to target regions in exons 1, 4, 5, 7, 9, and 12. Exon 1 contains the initiation codon, and the skipping of 5, 7, 9, and 12 exons would induce a frameshift and generate a premature stop codon (in exon 13, 9, 10/11, and 12, respectively). Inducing exon 4 skipping does not change the reading frame; however, it is structurally important [5]. Furthermore, two amino acids, serine 113 and threonine 123, that are known to be phosphorylated in AD, are found within exon 4, providing support for the importance of exon 4 skipping [42,43]. AOs were designed to target these regions and block the binding of splicing factors. AOs that targeted regions in exon 4 (AO4 and AO5) and exon 5 (AO6) induced exon-skipping, but also showed downregulation of the *MAPT* transcript simultaneously. The full-length product and exon-skipped RT-PCR products from AO4-treated samples were sequenced to confirm that the exon-skipped product is missing exon 4 (Figure S11, Supplementary Material). AO that targeted exon 12 (AO19) resulted in downregulation of the *MAPT* transcript, but was not dose-dependent. AO4, AO5, and AO6 were designed to target the splicing enhancer region in the *MAPT* isoform, variant 2, and therefore induce exon 4 and exon 5 skipping. Although exon skipping and downregulation of the full-length product was observed in AO4-, AO5-, and AO6-treated cells, the increase of exon-skipped product identified was not equivalent to the reduction in the full-length product (Figure S4, Supplementary Material), indicating that exon skipping only accounts for part of the loss of full-length products. On the other hand, whilst AO19 induced a significant knockdown of the full-length product, it did not lead to visible exon skipping. Therefore, we speculate that in addition to exon skipping-mediated downregulation of the full-length product, the steric bulkiness imposed by the AOs that prevents the action of translational machinery and the non-sense mediated decay (NMD) of mRNA resulting from exon skipping-induced premature termination codons are also possible mechanisms for *MAPT* knockdown. The AOs in this study were compared to a scrambled control sequence, where the scrambled sequence was an AO with a random nucleotide sequence that did not bind through complementary base pairing to any known gene used in our studies. However, at 400 nM concentrations and higher, the scrambled control sequence seems to be toxic to the cells, possibly due to off-target effects. However, this off-target effect at a 400 nM concentration may also be due to the toxicity of having a 2’-OMePS backbone. This off-target effect may explain the reduced *MAPT* transcript expression level compared to *MAPT* transcript expression in untreated cells only observed at a 400 nM concentration (Figure 4C), and not at a 50 nM concentration (Figure 4D).

The most effective AO candidate, AO4 targeting exon 4, was found to be highly efficient in downregulating the *MAPT* transcript compared to the lead DNAzyme candidate RNV563, which was only found to be efficient at higher concentrations (58% knockdown at 400 nM), whereas AO4 was very effective, even at a concentration as low as 6.25 nM (>60% knockdown). The lead AO and DNAzyme candidates, AO4 and RNV563, were both further validated by testing the tau protein levels in SH-SY5Y cells, which demonstrated a reduction in the tau level at 24 and 48 h, and this effect lasted at least 6 days after transfection with AO4, but not with RNV563. Tau has a relatively long half-life of about 12–14 h [44], explaining why the inhibition is seen at 24 and 48 h after AO4 and RNV563 treatment (Figure 6). However, different from AO4, which recorded a gradual increase in tau protein suppression, after 6 days of treatment of RNV563, the tau protein expression level displayed dramatic variation between different tests. This phenomenon is probably because of the use of P3000 reagent during the transfection of RNV563. As previously reported [45], the application of P3000, whilst ensuring a better transfection of DNAzyme, comes with the problem of an increased cytotoxicity, especially for long-term treatment such as six days. The observed cytotoxicity may have affected the quality of protein extraction and resulted in unreliable western blotting results, as shown in Figure S8 of Supplementary Material. Treatment of AO4 also resulted in a shorter protein product after 24 and 48 h of transfection that was around 48 kDa (around 6–7 kDa shorter than the full-length product) (Figure 5). The shorter protein product does not correspond to the exon-skipped product as the exon-skipped product should only be 2–3 kDa smaller than the full-length product. However, it may correspond to the exon-skipped product if the larger difference in size may be attributed to the loss of post-translational modifications, in addition to the amino acids skipped from the full-length product. The AO4 needs to be further validated and its effect on existing neurofibrillary tangles needs to be tested.

## 5. Conclusions

In summary, we have successfully identified an arm-loop-arm-type DNAzyme, RNV563, with a 10–23 catalytic motif that cleaves the *MAPT* transcript both in vitro and in SH-SY5Y cells, by screening different DNAzyme constructs targeting various exons of the *MAPT* gene transcript. The DNAzyme, RNV563, can downregulate the tau protein by more than 50%. We have also identified a 2’-OMePS antisense oligonucleotide, AO4, that induces efficient exon 4 skipping and downregulates the *MAPT* transcript in SH-SY5Y cells at concentrations as low as 6.25 nM, and downregulates the tau protein by more than 70%. We believe that our findings will contribute towards tau targeting drug development and help to improve the knowledge in the field. 

## Figures and Tables

**Table 1:** Second-generation DNAzymes derived from the first-generation parent DNAzyme RNV563, their sequences, and their target sequence.

Name	Sequence 5’→3’	Target Sequence
RNV563	GTTTATGA GGCTAGCTACAACGA GGATGTT	AACATCCATCATAAAC
RNV608	TTTATGA GGCTAGCTACAACGA GGATGT	ACATCCATCATAAA
RNV609	TTATGA GGCTAGCTACAACGA GGATG	CATCCATCATAA
RNV610	GGTTTATGA GGCTAGCTACAACGA GGATGTTG	CAACATCCATCATAAACC
RNV611	TGGTTTATGA GGCTAGCTACAACGA GGATGTTGC	GCAACATCCATCATAAACCA
RNV612	CTGGTTTATGA GGCTAGCTACAACGA GGATGTTGCC	GGCAACATCCATCATAAACCAG

## Supplementary Materials

The following are available online at https://www.mdpi.com/2073-4425/11/6/667/s1; Table S1. List of first-generation DNAzymes and their sequences; Figure S1. Representative RT-PCR products of the *MAPT* and *GAPDH* transcripts from SH-SY5Y cells after treatment with DNAzyme at 400 and 50 nM concentrations; Figure S2. Representative RT-PCR products of the *MAPT* and *GAPDH* transcripts from SH-SY5Y cells after treatment with DNAzyme at 400, 200, 100, and 50 nM concentrations; Figure S3. Representative RT-PCR products of the *MAPT* and *GAPDH* transcripts from SH-SY5Y cells after treatment with DNAzyme at 400, 200, 100, and 50 nM concentrations; Table S2. The average activity of second-generation DNAzymes (at a 400 nM concentration) in SH-SY5Y cells (knockdown of the *MAPT* transcript); Figure S4. In vitro cleavage of the FAM-conjugated *MAPT* RNA template composed of the exon 11 region (34 nucleotides) by RNV563 and its derivatives; Table S3. List of 2’-OMePS AOs and their sequences; Figure S5. Representative RT-PCR products of the *MAPT* transcripts from SH-SY5Y cells after treatment with splice-modulating AOs at 400 and 50 nM concentrations; Figure S6. A. Representative RT-PCR products of the *MAPT* transcripts from SH-SY5Y cells after treatment with AO4, AO5, AO6, and AO19 at 400, 200, 100, and 50 nM concentrations. B. Representative RT-PCR products of the *MAPT* and *GAPDH* transcripts from SH-SY5Y cells after treatment with AO4, AO5, AO6, and AO19 at 50, 25, 12.5, and 6.25 nM concentrations; Figure S7. A. Representative RT-PCR products of the *MAPT* transcripts from SH-SY5Y cells after treatment with an AO4 50 nM concentration and incubation of AO for 24 h, 48 h, and six days. B. Representative protein products of the *MAPT* and *GAPDH* transcripts from SH-SY5Y cells after treatment with AO4 at 50 nM concentrations and incubation of AO for 24 h, 48 h, and six days; Figure S8. A. Representative protein products of the *MAPT* and β-actin transcripts from SH-SY5Y cells after treatment with AO4 at 50 nM concentrations and incubation of AO for 24 h, 48 h, and six days. AO4 targets exon 4 of the *MAPT* transcript. The scrambled control was used at a 50 nM concentration and samples were collected after six days of transfection. B. Representative protein products of the *MAPT* and β-actin transcripts from SH-SY5Y cells after treatment with RNV563 at 400 nM concentrations and incubation of DNAzyme for 24 h, 48 h, and six days. RNV563 targets exon 9 of the *MAPT* transcript. The scrambled control was used at a 50 nM concentration and samples were collected after six days of transfection; Table S4. List of primers used, their sequences, and the expected product lengths; Table S5. The PCR conditions for each primer set; Table S6. List of primers used, their binding co-ordinates, the expected full-length product size, and the exon-skipped product size; Figure S9. Exon map of the *MAPT* variants found in the human brain and the primer binding sites of the primer sets used in this study; Figure S10. Sequence alignment of the full-length band (968 bp product) from RNV563-treated samples and the longest *MAPT* isoform, variant 2; Figure S11. A. Exon 3-Exon 5 region of the sequencing of the FL (307 bp) product from the AO4-treated sample. B. Exon 3-Exon 5 region of the sequencing of the exon-skipped (241 bp) product from the AO-treated sample.
