# Supplementary material for: Development of Novel Chemically-Modified Nucleic Acid Molecules for Efficient Inhibition of Human MAPT Gene Expression
Source: Genes (Basel). 2020 Jun 19;11(6):667. doi: 10.3390/genes11060667 (PMC7349501; doi:10.3390/genes11060667)
Supplement: Supplementary file 1 [file genes-11-00667-s001.pdf]

Supplementary Material: Development of novel chemically-modified nucleic acid molecules for efficient inhibition of human *MAPT* gene expression

Madhuri Chakravarthy, Suxiang Chen, Tao Wang and Rakesh N. Veedu

Table S1. List of first-generation DNAzymes and their sequences. Red nucleotides represent nucleotides in the catalytic loop. Blue nucleotides represent the cleavage site. Blue underlined nucleotides represent the cleavage site on the target sequence.

Name	Sequence	Target Sequence	Target
**RNV547**	ATCTTCCA GGCTAGCTACAACGA CACTTCG	CGAAGTGATGGAAGAT	Exon 1
**RNV548**	CAGCGTGA GGCTAGCTACAACGA CTTCCAT	ATGGAAGATCACGCTG	Exon 1
**RNV549**	CCCCCTGA GGCTAGCTACAACGA CTTTCCT	AGGAAAGATCAGGGGG	Exon 1
**RNV550**	TTGGTGCA GGCTAGCTACAACGA GGTGTAG	CTACACCATGCACCAA	Exon 1
**RNV551**	TCCTCAGA GGCTAGCTACAACGA CCGTCCT	AGGACGGATCTGAGGA	Exon 2
**RNV552**	TCTTAGCA GGCTAGCTACAACGA CAGAGGT	ACCTCTGATGCTAAGA	Exon 2
**RNV553**	CTCCCTCA GGCTAGCTACAACGA CCACTAA	TTAGTGGATGAGGGAG	Exon 3
**RNV554**	TTCTGGGA GGCTAGCTACAACGA CTCCGTG	CACGGAGATCCCAGAA	Exon 3
**RNV555**	GTCTCCAA GGCTAGCTACAACGA GCCTGCT	AGCAGGCATTGGAGAC	Exon 3
**RNV556**	TTTTGTCA GGCTAGCTACAACGA CGCTTCC	GGAAGCGATGACAAAA	Exon 5
**RNV557**	TGTGGCGA GGCTAGCTACAACGA CTTCGTT	AACGAAGATCGCCACA	Exon 7
**RNV558**	TGCTGGAA GGCTAGCTACAACGA CCTGGTG	CACCAGGATTCCAGCA	Exon 7
**RNV559**	TCCCCTGA GGCTAGCTACAACGA TTTGGAG	CTCCAAAATCAGGGGA	Exon 9

**RNV560**	CCGCTGCGA<br>GGCTAGCTACAACGA<br>CCCCTGA	TCAGGGGATCGCAGCGG	Exon 9
**RNV561**	ACTTGACA<br>GGCTAGCTACAACGA<br>TCTTCAG	CTGAAGAATGTCAAGT	Exon 9
**RNV562**	TTG CCTAA<br>GGCTAGCTACAACGA<br>GAGCCAC	GTGGCTCATTAGGCAA	Exon 11
**RNV563**	GTTTATGA<br>GGCTAGCTACAACGA<br>GGATGTT	AACATCCATCATAAAC	Exon 11
**RNV564**	CTGGTTTA<br>GGCTAGCTACAACGA<br>GATGGAT	ATCCATCATAAACCAG	Exon 11
**RNV565**	TTCTCAGA<br>GGCTAGCTACAACGA<br>TTTACTT	AAGTAAAATCTGAGAA	Exon 12
**RNV566**	GGACCCAA<br>GGCTAGCTACAACGA<br>CTTCGAC	GTCGAAGATTGGGTCC	Exon 12
**RNV567**	GGGTGATA<br>GGCTAGCTACAACGA<br>TGTCCAG	CTGGACAATATCACCC	Exon 12
**RNV568**	GTGGGTGA<br>GGCTAGCTACAACGA<br>ATTGTCC	GGACAATATCACCCAC	Exon 12
**RNV569**	GTACACGA<br>GGCTAGCTACAACGA<br>CTCCGCC	GGCGGAGATCGTGTAC	Exon 13
**RNV570**	TGCTGAGA<br>GGCTAGCTACAACGA<br>GCCGTGG	CCACGGCATCTCAGCA	Exon 13
**RNV571**	AGGAGACA<br>GGCTAGCTACAACGA<br>TGCTGAG	CTCAGCAATGTCTCCT	Exon 13
**RNV572**	CATGTCGA<br>GGCTAGCTACAACGA<br>GCTGCCG	CGGCAGCATCGACATG	Exon 13
**RNV573**	GTCTACCA<br>GGCTAGCTACAACGA<br>GTCGATG	CATCGACATGGTAGAC	Exon 13

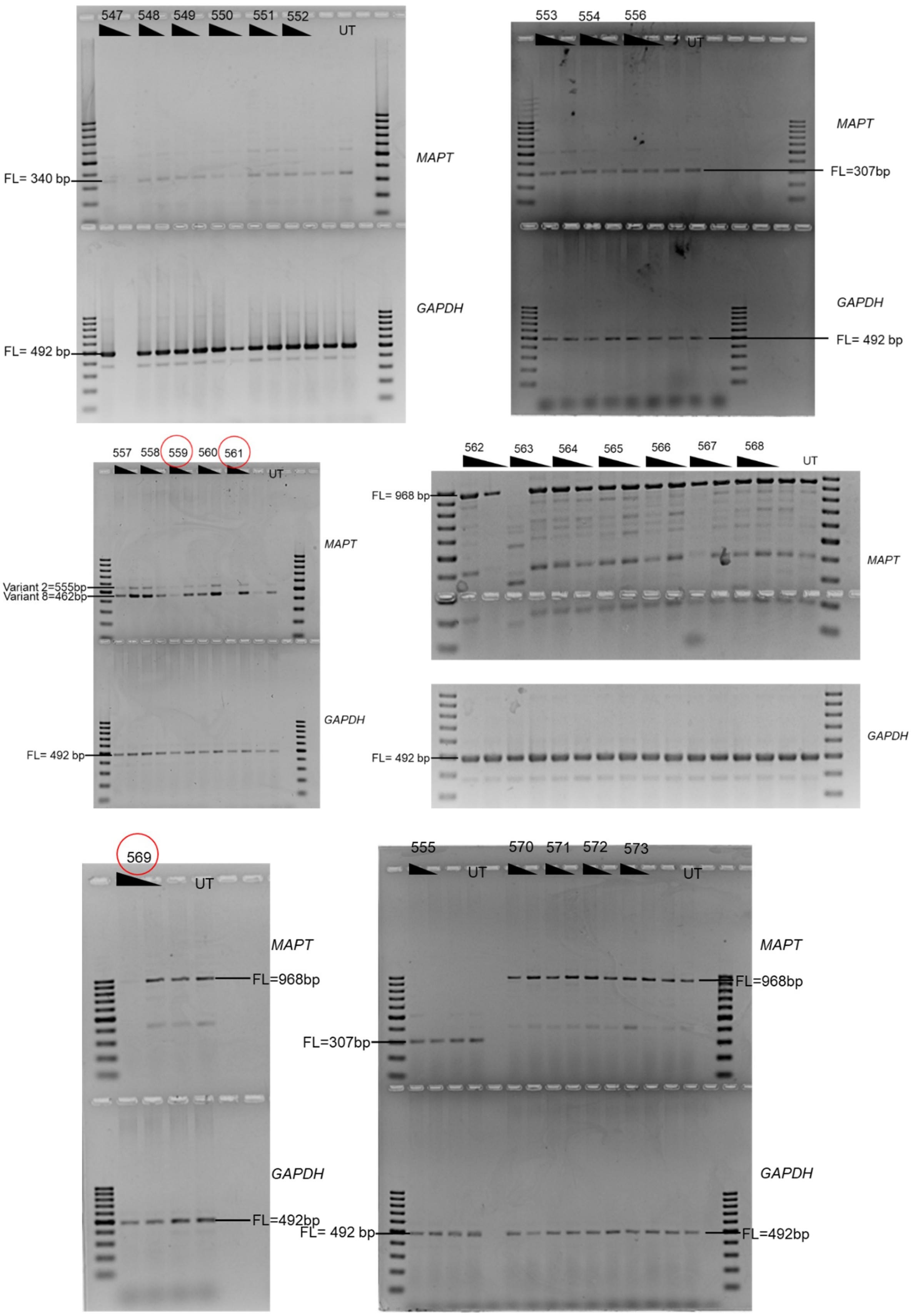

Figure S1: Representative RT-PCR products of the *MAPT* and *GAPDH* transcripts from SH-SY5Y cells after treatment with DNAzyme at 400 nM, and 50 nM concentrations. The RT-PCR products after treatment with RNV547, RNV548, RNV549, RNV550, RNV551, RNV552, RNV553, RNV554, RNV555, RNV556, RNV557, RNV558, RNV559, RNV560, RNV561, RNV562, RNV563, RNV564, RNV565, RNV566, RNV567, RNV568, RNV569, RNV570, RNV571, RNV572 and RNV573 are shown here. The

RNA from RNV547- RNV552 treated samples were amplified using primer set 1. The RNA from RNV553-RNV556 treated samples were amplified using primer set 2. The RNA from RNV557-561 treated samples were amplified using primer set 3. The RNA from RNV562-RNV573 treated samples were amplified using primer set 4. RNV FL, full-length; UT, untreated; *GAPDH* was used as a loading control.

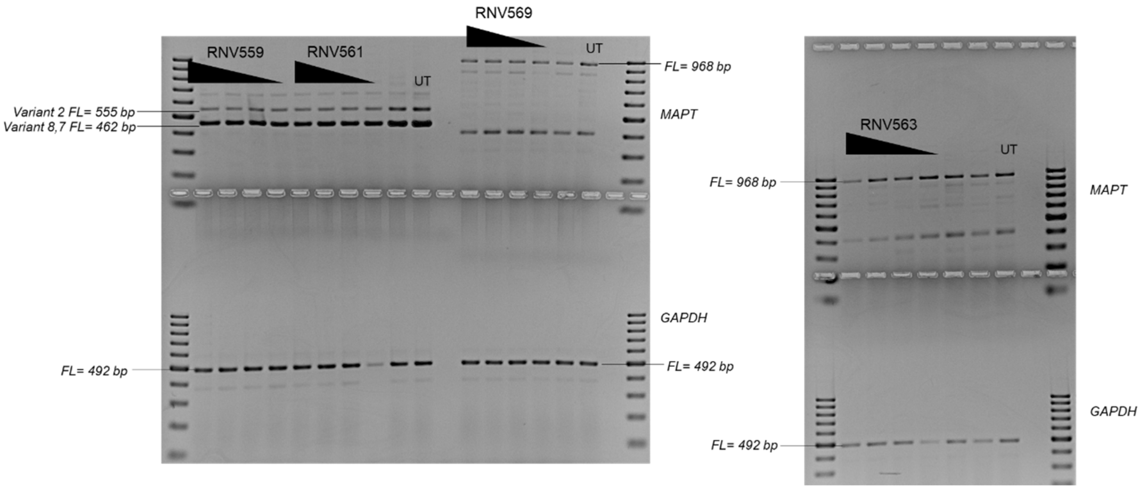

Figure S2: Representative RT-PCR products of the *MAPT* and *GAPDH* transcripts from SH-SY5Y cells after treatment with DNAzyme at 400 nM, 200 nM, 100 nM and 50 nM concentrations. The RT-PCR products after treatment with RNV559, RNV561, RNV563 and RNV563 are shown here. The RNA from RNV559 and RNV561 treated samples were amplified using primer set 2. The RNA from RNV563 and RNV569 treated samples were amplified using primer set 4. FL, full-length; UT, untreated; *GAPDH* was used as a loading control. [The gel in this figure is the original gel representing the gel in Figure 2 of the article. The cropped gel has been shown in Figure 2 of the article due to other unimportant samples that exist between the desired samples.].

Table S2: The average activity of 1st generation DNAzymes (at 400 nM concentration) in SH-SY5Y cells (knockdown of *MAPT* transcript).

Name	Average Activity of DNAzyme in SH-SY5Y cells
**559**	0%
**561**	26%
**563**	58%
**569**	0%

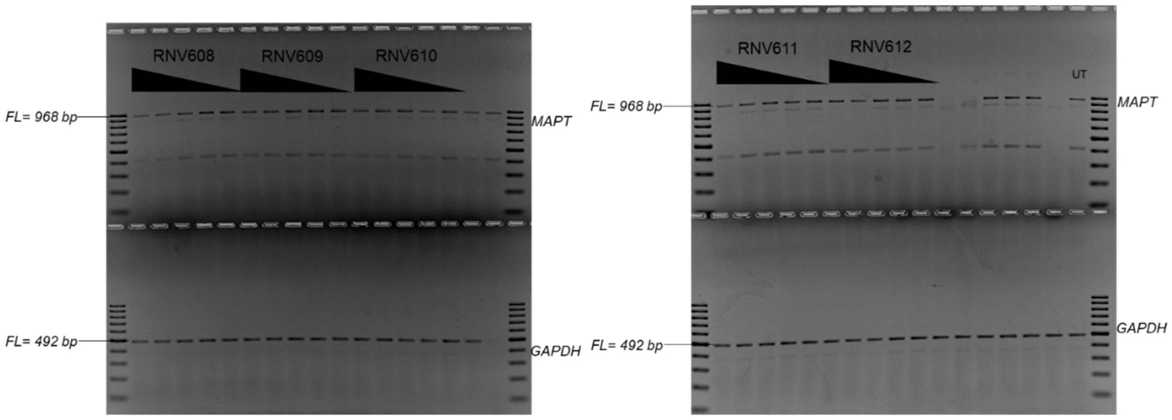

Figure S3: Representative RT-PCR products of the *MAPT* and *GAPDH* transcripts from SH-SY5Y cells after treatment with DNAzyme at 400 nM, 200 nM, 100 nM and 50 nM concentrations. The RT-PCR

products after treatment with RNV608, RNV609, RNV610, 611 and RNV612 are shown here and were amplified using primer set 4. FL, full-length; UT, untreated; *GAPDH* was used as a loading control.

Table S3. The average activity of 2nd generation DNAzymes (at 400 nM concentration) in SH-SY5Y cells (knockdown of *MAPT* transcript).

Name	Average Activity of DNAzymes in SH-SY5Y cells
**608**	0%
**609**	0%
**610**	15%
**611**	0%
**612**	2%

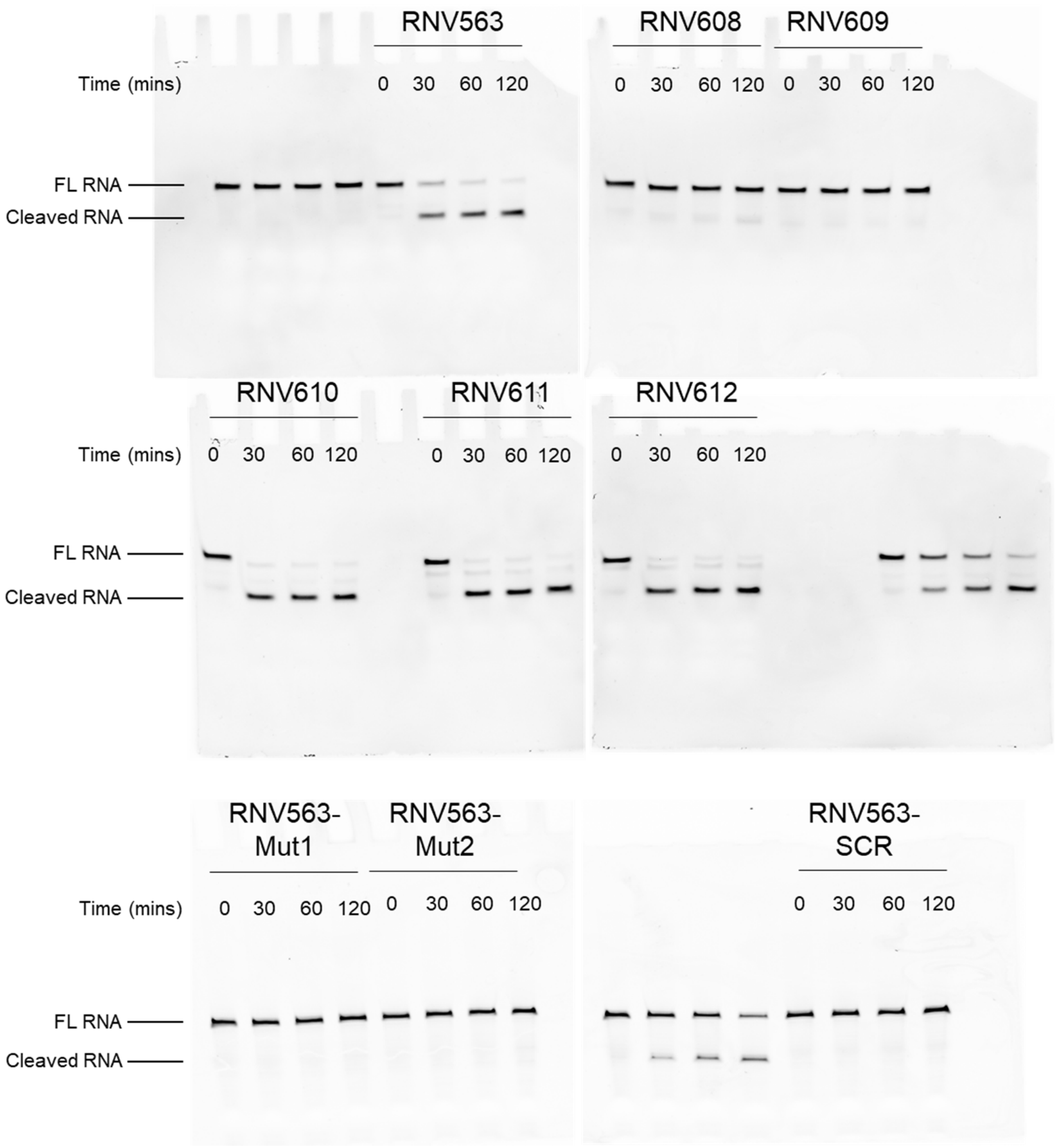

Figure S4. *In vitro* cleavage of the FAM-conjugated *MAPT* RNA template composed of exon 11 region (34 nucleotides) by RNV563 and its derivatives. FL RNA, full-length; FAM-conjugated RNA; cleaved RNA; the cleaved FAM-conjugated *MAPT* RNA (22 nucleotides long). The FAM- conjugated template RNA is a small region of the *MAPT* transcript complementary to the hybridisation arms of the DNAzymes of interest. [The gel in this figure is the original gel representing the gel in Figure 3 of the article. The cropped gel has been shown in Figure 3 of the article due to other unimportant samples or unwanted spaces that exist between the desired samples.].

Table S4. List of 2′-OMePS AOs and their sequences. In the sequences, capital letters denote bases from the exon regions, and small letters denote bases from the intronic region.

AO Number	AO Name	AO Sequence	Target
**AO1**	MAPT E1A(+11+35)	TCCATCACTTCGAACTCCTGGCGGG	Exon 1
**AO2**	MAPT E1A(+41+65)	TCCCCCAACCCGTACGTCCCAGCGT	Exon 1
**AO3**	MAPT E1A(+91+115)	TGTCACCCTCTTGGTCTTGGTGCAT	Exon 1
**AO4**	MAPT E4A(+1+25)	GTGTCTCCAATGCCTGCTTCTTCAG	Exon 4
**AO5**	MAPT E4A(+26+50)	AGCAGCTTCGTCTTCCAGGCTGGGG	Exon 4
**AO6**	MAPT E5A(+16+40)	TCGCTTCCAGTCCCGTCTTTGCTTT	Exon 5
**AO7**	MAPT E5D(+3-22)	ctccgtggcatcgtcagcttacCTT	Exon 5
**AO8**	MAPT E7A(+26+50)	GGAGGGGCTGCTCCCCGCGGTGTGG	Exon 7
**AO9**	MAPT E7A(+46+70)	TGGCCTGGCCCTTCTGGCCTGGAGG	Exon 7
**AO10**	MAPT E7A(+71+95)	GTTTTTGCTGGAATCCTGGTGGCGT	Exon 7
**AO11**	MAPT E7A(+97+121)	TGGGTGGTGTCTTTGGAGCGGGCGG	Exon 7
**AO12**	MAPT E7D(+5-20)	caagagaacgttcttcttacCAGAG	Exon 7
**AO13**	MAPT E9A(+1+25)	CGATCCCCTGATTTTGGAGGTTCAC	Exon 9
**AO14**	MAPT E9A(+111+135)	TACGGACCACTGCCACCTTCTTGGG	Exon 9
**AO15**	MAPT E9A(+159+183)	GGGCTGTCTGCAGGCGGCTCTTGGC	Exon 9
**AO16**	MAPT E9A(+196+220)	TTGGACTTGACATTCTTCAGGTCTG	Exon 9
**AO17**	MAPT E9A(+226+250)	TGGTGCTTCAGGTTCTCAGTGGAGC	Exon 9
**AO18**	MAPT E9D(+21-4)	tcacCTTCCCGCCTCCCGGCTGGTG	Exon 9
**AO19**	MAPT E12A(-5+20)	TACTTCCACCTGGCCACCTCctaga	Exon 12
**AO20**	MAPT E12A(+21+45)	CCTTGAAGTCAAGCTTCTCAGATTT	Exon 12
**AO21**	MAPT E12A(+46+70)	GACCCAATCTTCGACTGGACTCTGT	Exon 12
**AO22**	MAPT E12A(+81+95)	AGGGACGTGGGTGATATTGTCCAGG	Exon 12

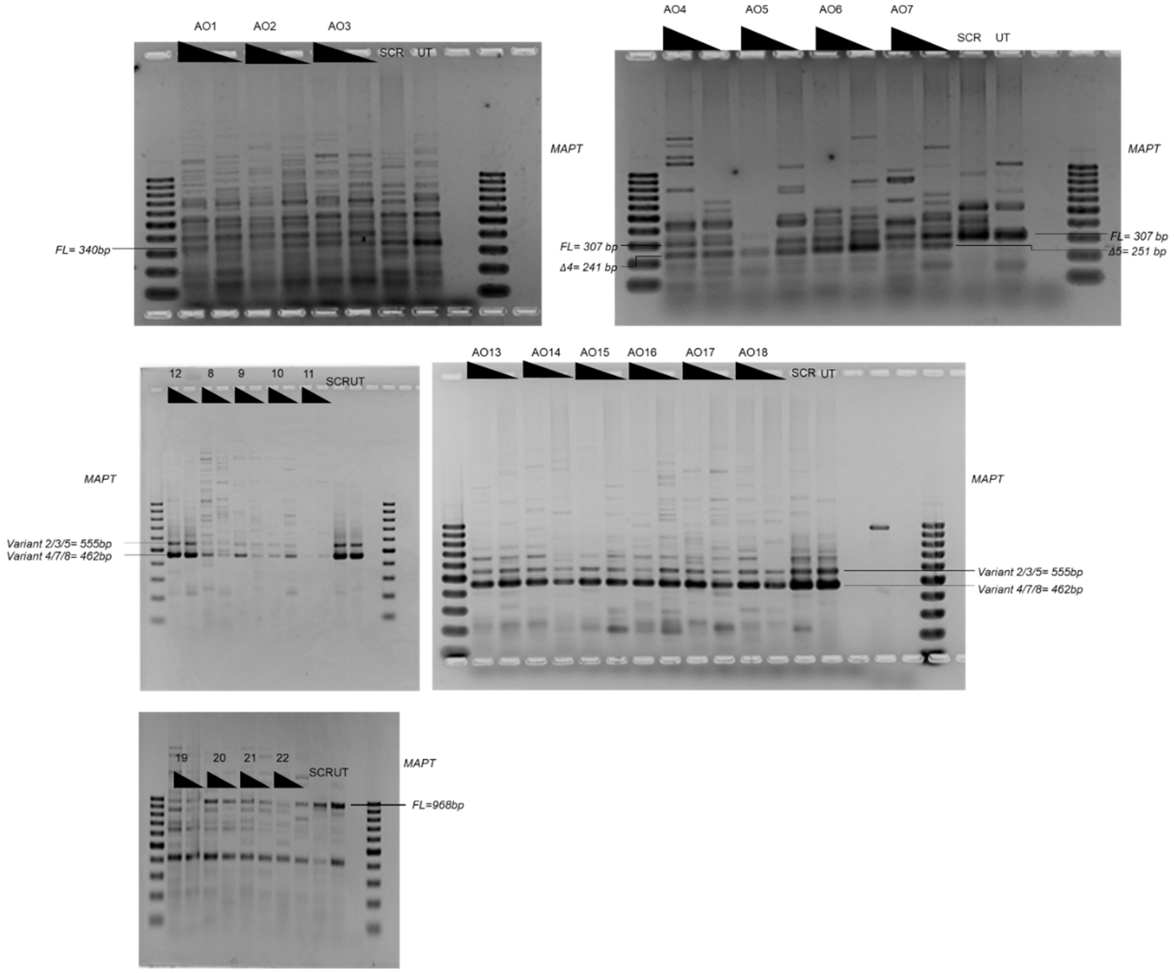

Figure S5: Representative RT-PCR products of the *MAPT* transcripts from SH-SY5Y cells after treatment with splice-modulating AOs at 400 nM, and 50 nM concentrations. The RT-PCR products after treatment with AOs 1-22 are shown here. The RNA from AOs 1-3 were amplified using primer set 2. The RNA from AOs 4-18 were amplified using primer set 3. The RNA from AOs 19-22 were amplified using primer set 4. FL, full-length; UT, untreated; SCR, scrambled sequence.

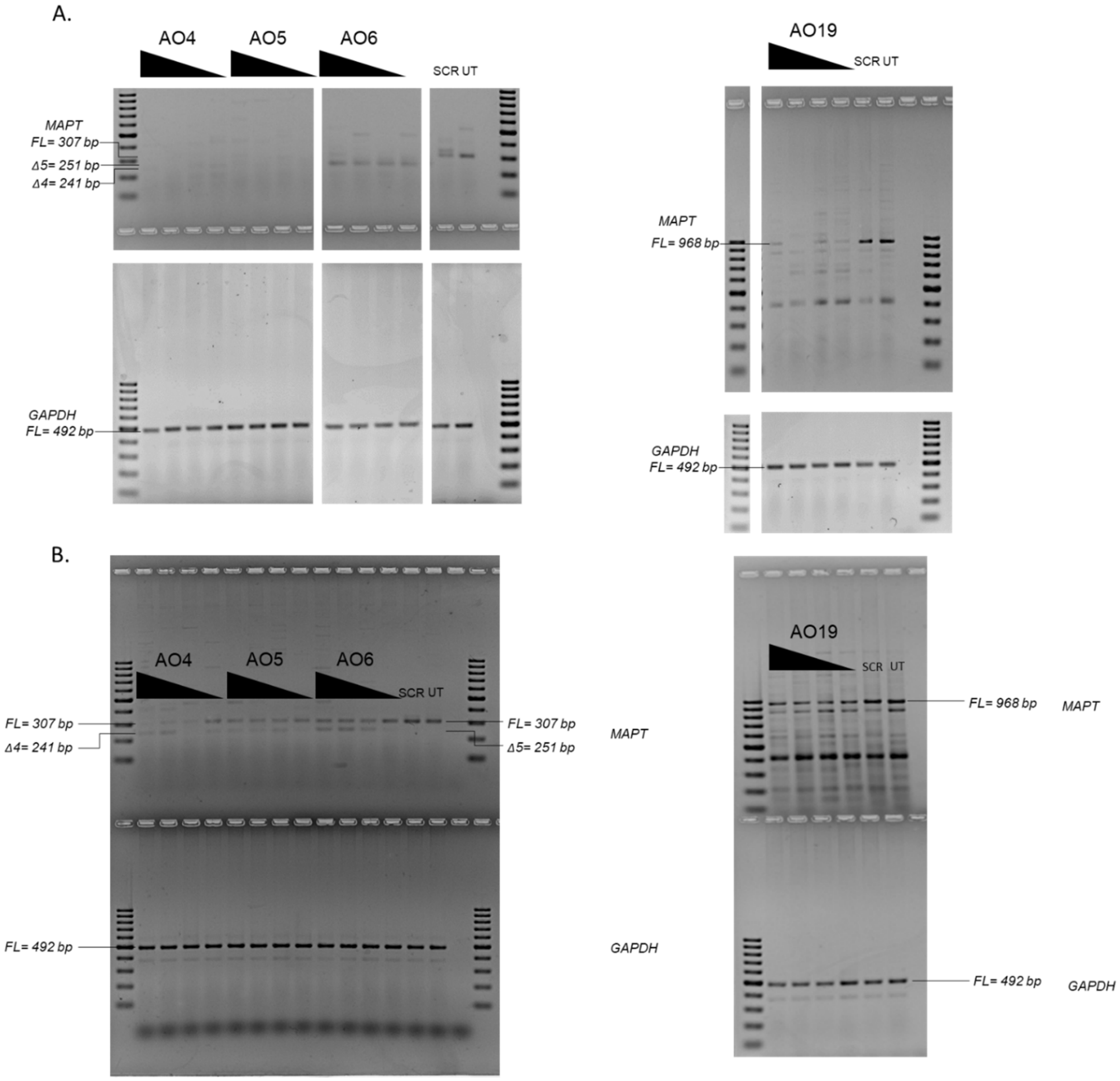

Figure S6. A. Representative RT-PCR products of the *MAPT* transcripts from SH-SY5Y cells after treatment with AO4, AO5, AO6, and AO19 at 400 nM, 200 nM, 100 nM and 50 nM concentrations. B. Representative RT-PCR products of the *MAPT* and *GAPDH* transcripts from SH-SY5Y cells after treatment with AO4, AO5, AO6, and AO19 at 50 nM, 25 nM, 12.5 nM and 6.25 nM concentrations. AO4 targets exon 4, AO5 and AO6 target exon 5, and AO19 targets exon 12 of the *MAPT* transcript. The RNA form AO4, AO5 and AO6 treated samples were amplified using primer set 2. The RNA from AO19 treated samples were amplified using primer set 4. FL, full-length; UT, untreated; SCR, scrambled sequence; *GAPDH* was used as a loading control. [The gel in this figure is the original gel representing the gel in Figure 4 of the article. The cropped gel has been shown in Figure 4 of the article due to unwanted spaces that exist on the gel.].

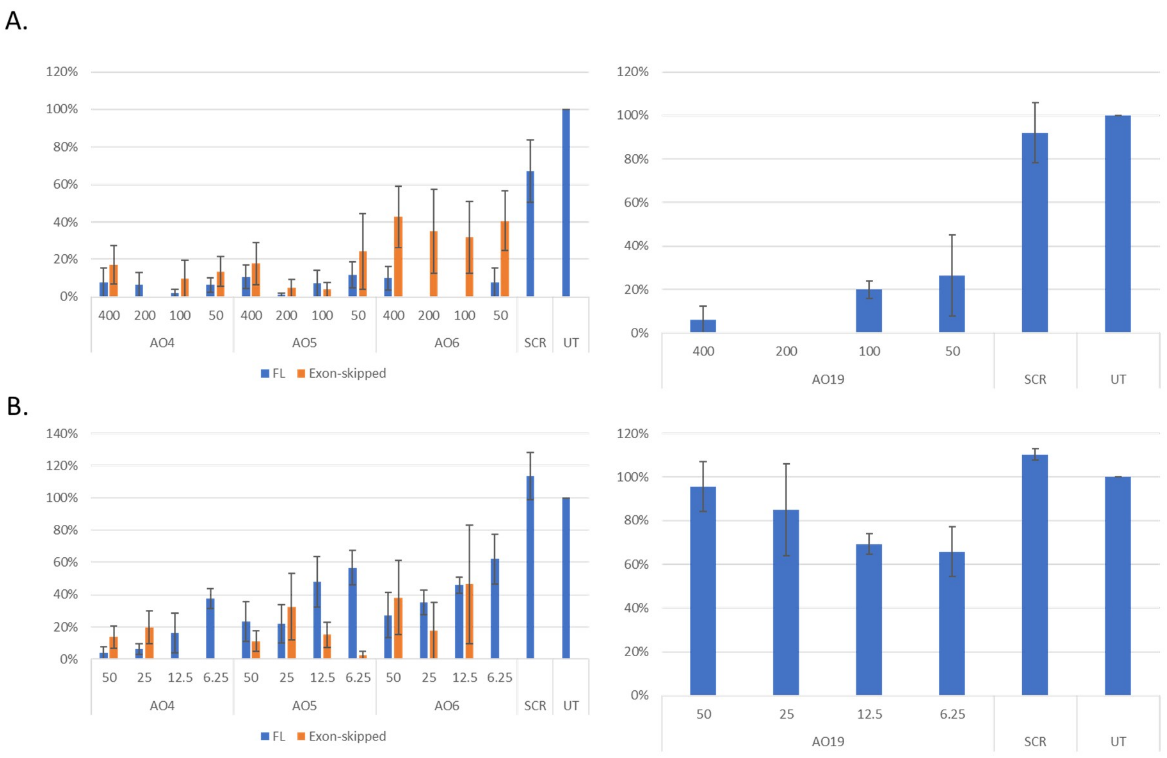

Figure S7. Densitometry analysis of RT-PCR products (three replicates) using AO4, AO6, AO7 and AO19 showed downregulation and exon-skipping of *MAPT* transcript in SH-SY5Y cells *in vitro*. Concentrations of AOs used include 400 nM, 200 nM, 100 nM and 50 nM. The error bars represent the standard error of mean. B. Densitometry analysis of RT-PCR products (more than two replicates) using AO4, AO6, AO7 and AO19 showed downregulation and exon-skipping of *MAPT* transcript in SH-SY5Y cells *in vitro*. Concentrations of AOs used include 50 nM, 25 nM, 12.5 nM and 6.25 nM. The error bars represent the standard error of mean.

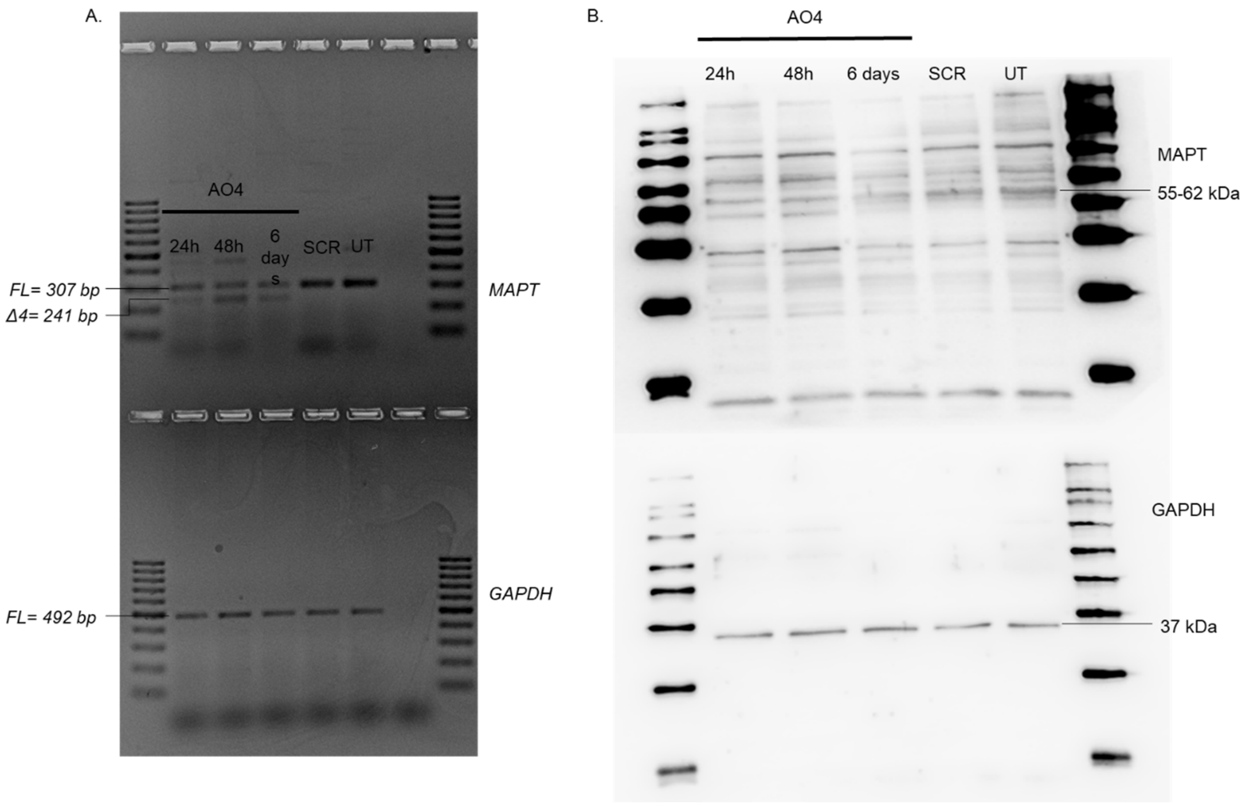

Figure S8. A. Representative RT-PCR products of the MAPT transcripts from SH-SY5Y cells after treatment with AO4 50 nM concentration and incubation of AO for 24 h, 48 h and six days. AO4 targets exon 4 of the *MAPT* transcript. **B**. Representative protein products of the *MAPT* and *GAPDH* transcripts from SH-SY5Y cells after treatment with AO4 at 50 nM concentrations and incubation of AO for 24 h, 48 h and six days. AO4 targets exon 4 of the *MAPT* transcript. The RNA from AO4 treated samples for the RT-PCR were amplified using primer set 2. FL, full-length; UT, untreated; SCR, scrambled sequence; *GAPDH* was used as a loading control. [The gel in this figure is the original gel representing the gel in Figure 5 of the article. The cropped gel has been shown in Figure 5 of the article due to unwanted spaces, other unimportant samples, and non-specific bands that exist on the gel and membrane.].

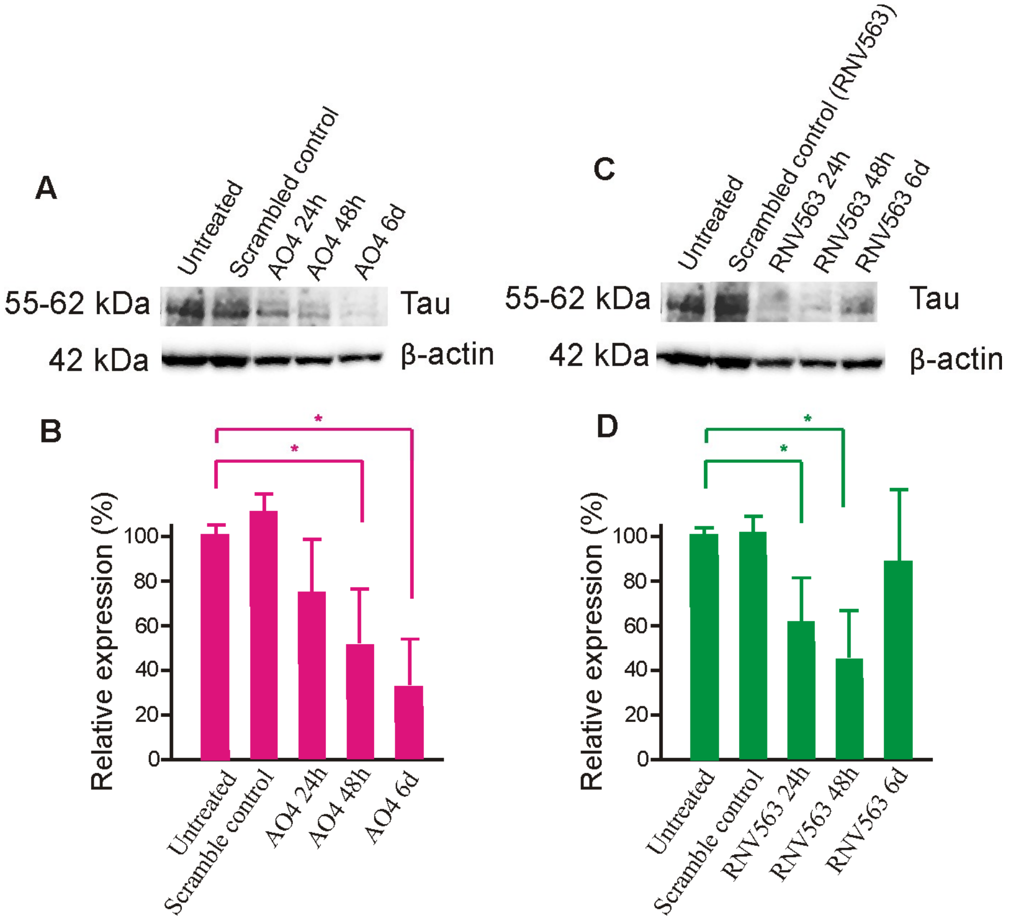

Figure S9. A. Representative protein products of the *MAPT* and *β-actin* transcripts from SH-SY5Y cells after treatment with AO4 at 50 nM concentrations and incubation of AO for 24 h, 48 h and six days. AO4 targets exon 4 of the *MAPT* transcript. **B**. Densitometry analysis of the western blot (three replicates) shown in A of this figure. The error bars represent the standard error of mean. **C**. Representative protein products of the *MAPT* and *β-actin* transcripts from SH-SY5Y cells after treatment with RNV563 at 400 nM concentrations and incubation of DNAzyme for 24 h, 48 h and six days. RNV563 targets exon 9 of the *MAPT* transcript. **D**. Densitometry analysis of the western blot (three replicates) shown in C of this figure. The error bars represent the standard error of mean.

Table S5. List of primers used, their sequences and the expected product lengths.

Primer Set	Primer pairs	Primer Sequences	Expected product length
**MAPT Primer Set 1**	MAPT6_Ex0Fa<br>MAPT6_Ex3R	5′ TCCTCGCCTCTGTCGACTAT 3′<br>5′ TCCTTCTGGGATCTCCGTGT 3′	Variant 2/8= 340 bp

**MAPT Primer Set 2**	MAPT6_Ex3F<br>MAPT6_Ex7R	5′ GTGACAGCACCCTTAGTGGA 3′<br>5′ GCGGGGTTTTTGCTGGAATC 3′	Variant 2/8= 307 bp
**MAPT Primer Set 3**	MAPT6_Ex5F<br>MAPT6_Ex11R	5′ AAGACGGGACTGGAAGCGAT 3′<br>5′ TGCTCAGGTCAACTGGTTTGT 3′	Variant 2/3/5= 555 bp<br>Variant 4/7/8= 462 bp
**MAPT Primer Set 4**	MAPT6_Ex10F<br>MAPT6_Ex13Ra	5′ TTAGCAACGTCCAGTCCAAGT 3′<br>5′ AGGTTGACATCGTCTGCCTG 3′	Variant 2/3/5= 968 bp
**GAPDH Primer set**	GAPDH For<br>GAPDH Rev	5′ GGACTCATGACCACAGTCCATGC 3′<br>5′ TTACTCCTTGGAGGCCATGTGGG 3′	492 bp

Table S6. The PCR conditions for each primer set.

**MAPT Primer Set 1 (50 ng each)**	**Temperature**	**Time**	
	55°C	30 min	
	94°C	2 min	
	94°C	30 s	28 cycles
	55°C	1 min	
	68°C	2 min	
**MAPT Primer Set 3 & 4 (50 ng each)**	Temperature	Time	
	55°C	30 min	
	94°C	2 min	
	94°C	30 s	34 cycles
	60°C	1 min	
	68°C	2 min	
**MAPT Primer Set 2 & 3 (50 ng each)**	**Temperature**	**Time**	
	55°C	30 min	
	94°C	2 min	
	94°C	30 s	30 cycles
	60°C	1 min	
	68°C	2 min	
**GAPDH Primer set (12.5 ng each)**	**Temperature**	**Time**	
	55°C	30 min	
	94°C	2 min	
	94°C	30 s	16 cycles

Table S7. List of primers used, their binding co-ordinates, the expected full-length product size and the exon-skipped product size.

MAPT Primer Set	Primer pairs	Primer binding Co-ordinates	Expected full-length product size	Exon skipped product size
1	MAPT6_Ex0Fa<br>MAPT6_Ex3R	Exon 0 (+283 +302)<br>Exon 3 (+79+60)	Variant 2/8= 340 bp	ΔExon1=190bp
2	MAPT6_Ex3F<br>MAPT6_Ex7R	Exon 3 (+2+21)<br>Exon 7 (+99+80)	Variant 2/8= 307 bp	ΔExon4=241bp<br>ΔExon5=251bp
3	MAPT6_Ex5F<br>MAPT6_Ex11R	Exon 5 (+21+40)<br>Exon 11 (+33+13)	Variant 2/3/5= 555 bp<br>Variant 4/7/8= 462 bp	ΔExon7<br>Variant 2/3/5=428bp<br>Variant 4/7/8=335bp<br>ΔExon9<br>Variant 2/3/5=289bp

				Variant 4/7/8=196bp
**4**	MAPT6_Ex10F<br>MAPT6_Ex13Ra	Exon 10 (+28+48)<br>Exon 13 (+707+688)	Variant 2/3/5= 968 bp	ΔExon12=855bp

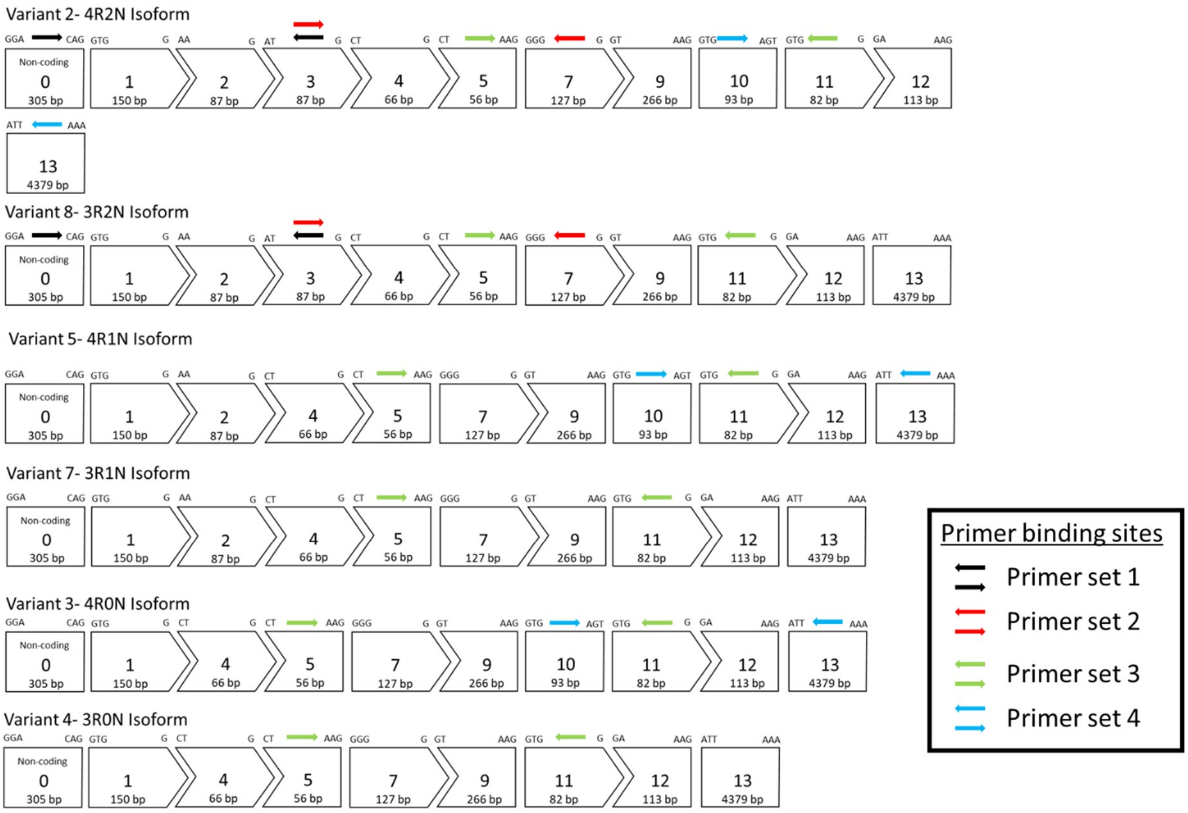

Figure S10. Exon map of the *MAPT* variants found in the human brain and the primer binding sites of the primer sets used in this study.

Score	Expect	Identities	Gaps	Strand
1482 bits(802)	0.0	811/818(99%)	1/818(0%)	Plus/Plus

```
Query  1259  GTTGACCTGAGCAAGGTGACCTCCAAGTGTGGCTCATTAGGCAACATCCATCATAAACCA  1318
             ||||||||||||||||||||||||||||||||||||||||||||||||||||||||||||
Sbjct  62    GTTGACCTGAGCAAGGTGACCTCCAAGTGTGGCTCATTAGGCAACATCCATCATAAACCA  121

Query  1319  GGAGGTGGCCAGGTGGAAGTAAAATCTGAGAAGCTTGACTTCAAGGACAGAGTCCAGTCG  1378
             ||||||||||||||||||||||||||||||||||||||||||||||||||||||||||||
Sbjct  122   GGAGGTGGCCAGGTGGAAGTAAAATCTGAGAAGCTTGACTTCAAGGACAGAGTCCAGTCG  181

Query  1379  AAGATTGGGTCCCTGGACAATATCACCCACGTCCCTGGCGGAGGAAATAAAAAGATTGAA  1438
             ||||||||||||||||||||||||||||||||||||||||||||||||||||||||||||
Sbjct  182   AAGATTGGGTCCCTGGACAATATCACCCACGTCCCTGGCGGAGGAAATAAAAAGATTGAA  241

Query  1439  ACCCACAAGCTGACCTTCCGCGAGAACGCCAAAGCCAAGACAGACCACGGGGCGGAGATC  1498
             ||||||||||||||||||||||||||||||||||||||||||||||||||||||||||||
Sbjct  242   ACCCACAAGCTGACCTTCCGCGAGAACGCCAAAGCCAAGACAGACCACGGGGCGGAGATC  301

Query  1499  GTGTACAAGTCGCCAGTGGTGTCTGGGGACACGTCTCCACGGCATCTCAGCAATGTCTCC  1558
             ||||||||||||||||||||||||||||||||||||||||||||||||||||||||||||
Sbjct  302   GTGTACAAGTCGCCAGTGGTGTCTGGGGACACGTCTCCACGGCATCTCAGCAATGTCTCC  361

Query  1559  TCCACCGGCAGCATCGACATGGTAGACTCGCCCCAGCTCGCCACGCTAGCTGACGAGGTG  1618
             ||||||||||||||||||||||||||||||||||||||||||||||||||||||||||||
Sbjct  362   TCCACCGGCAGCATCGACATGGTAGACTCGCCCCAGCTCGCCACGCTAGCTGACGAGGTG  421

Query  1619  TCTGCCTCCCTGGCCAAGCAGGGTTTGTGATCAGGCCCCTGGGGCGGTCAATAATTGTGG  1678
             ||||||||||||||||||||||||||||||||||||||||||||||||||||||||||||
Sbjct  422   TCTGCCTCCCTGGCCAAGCAGGGTTTGTGATCAGGCCCCTGGGGCGGTCAATAATTGTGG  481

Query  1679  AGAGGAGAGAATGAGAGAGTGTGGaaaaaaaaaGAATAATGACCCGGCCCCCGCCCTCTG  1738
             ||||||||||||||||||||||||||||||||||||||||||||||||||||||||||||
Sbjct  482   AGAGGAGAGAATGAGAGAGTGTGGAAAAAAAAAGAATAATGACCCGGCCCCCGCCCTCTG  541

Query  1739  CCCCCAGCTGCTCCTCGCAGTTCGGTTAATTGGTTAATCACTTAACCTGCTTTTGTCACT  1798
             ||||||||||||||||||||||||||||||||||||||||||||||||||||||||||||
Sbjct  542   CCCCCAGCTGCTCCTCGCAGTTCGGTTAATTGGTTAATCACTTAACCTGCTTTTGTCACT  601

Query  1799  CGGCTTTGGCTCGGGACTTCAAAATCAGTGATGGGAGTAAGAGCAAATTTCATCTTTCCA  1858
             ||||||||||||||||||||||||||||||||||||||||||||||||||||||||||||
Sbjct  602   CGGCTTTGGCTCGGGACTTCAAAATCAGTGATGGGAGTAAGAGCAAATTTCATCTTTCCA  661

Query  1859  AATTGATGGGTGGGCTAGTAATAAAATATTTaaaaaaaaaCATTCAAAAACATGGCCACA  1918
             ||||||||||||||||||||||||||||||||||||||||||||||||||||||||||||
Sbjct  662   AATTGATGGGTGGGCTAGTAATAAAATATTTAAAAAAAAACATTCAAAAACATGGCCACA  721

Query  1919  TCCAACATTTCCTCAGGCAATTCCTTTTGATTCTTTTTTCTTCCCCCTCCATGTAGAAGA  1978
             ||||||||||||||||||||||||||||||||||||||||||||||||||||||| || |
Sbjct  722   TCCAACATTTCCTCAGGCAATTCCTTTTGATTCTTTTTTCTTCCCCCTCCATGTANAANA  781

Query  1979  GGGAGAAGGAGAGGCTCTGAAAGCTGCTTCTGGGGGATTTCAAGGACTGGGGTGCCAA  2038
              ||||||||| |||||||||||||||||||||||||| ||||||||||||||||||||||
Sbjct  782   NGGAGAAGGANAGGCTCTGAAAGCTGCTTCTGGGGGANTTCAAGGACTGGGGTGCCAA  841

Query  2039  CCACCTCTGGCCCTGTTGTGGGGGTGTCACAGAGGCAG  2076
             ||||||||||||||||||| ||||||||||| ||||||
Sbjct  842   CCACCTCTGGCCCTGTTGT-GGGGTGTCACANAGGCAG  878
```

Figure S11. Sequence alignment of the full length band (968bp product) from RNV563 treated samples and the longest *MAPT* isoform, variant 2. .

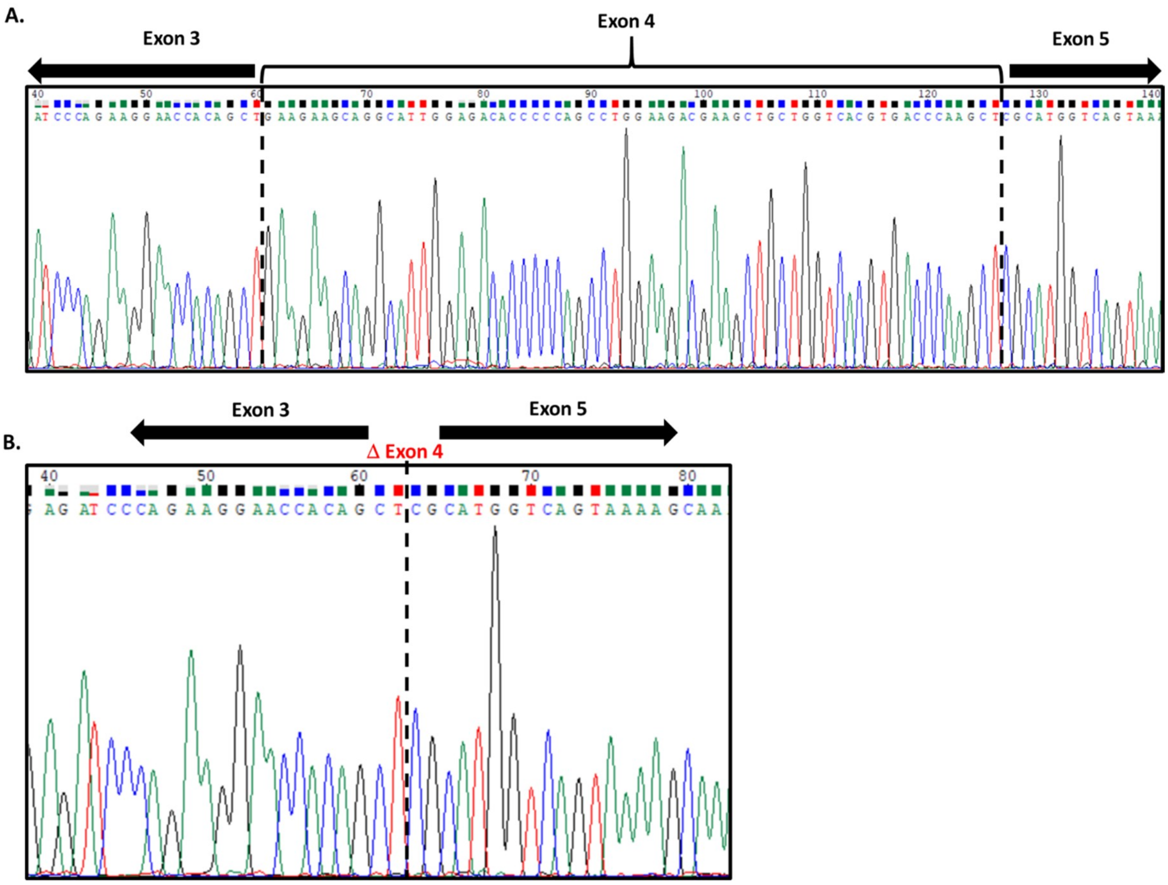

Figure S12. A. Exon 3- Exon 5 region of the sequencing of the FL (307 bp) product from AO4 treated sample. **B**. Exon 3- Exon 5 region of the sequencing of the exon-skipped (241 bp) product from AO treated sample.
